# Intergrative metabolomic and transcriptomic analyses unveil nutrient remobilization events in leaf senescence of tobacco

**DOI:** 10.1038/s41598-017-11615-0

**Published:** 2017-09-21

**Authors:** Wei Li, Hailiang Zhang, Xiaoxu Li, Fengxia Zhang, Cheng Liu, Yongmei Du, Xiaoming Gao, Zenglin Zhang, Xiaobing Zhang, Zhihui Hou, Hui Zhou, Xiaofei Sheng, Guodong Wang, Yongfeng Guo

**Affiliations:** 1 Tobacco Research Institute, Chinese Academy of Agricultural Sciences, Qingdao, Shandong 266101 China; 2 Institute of Genetics and Developmental Biology, Chinese Academy of Sciences, Datun Road, Chaoyang District, Beijing, 100101 China

## Abstract

Leaf senescence in plants is a coordinated process that involves remobilization of nutrients from senescing leaves to sink tissues. The molecular events associated with nutrient remobilization are however not well understood. In this study the tobacco system with a source-sink relationship between different leaf positions was used in analyzing the spatiotemporal changes of 76 metabolites from leaves at 3 different stalk positions and 8 developmental stages. The metabolomic data was then compared with RNA-seq data from the same samples to analyze the activities of the metabolic pathways that are important for nutrient remobilization. Integrative analyses on metabolites accumulation and expression changes of enzyme-encoding genes in corresponding metabolic pathways indicated a significant up-regulation of the tricarboxylic acid cycle and related metabolism of sugars, amino acids and fatty acids, suggesting the importance of energy metabolism during leaf senescence. Other changes of the metabolism during tobacco leaf senescence include increased activities of the GS/GOGAT cycle which is responsible for nitrogen recycling, and increased accumulation of nicotine. The results also suggested that a number of compounds seemed to be transported from senescing leaves at lower positions to sink leaves at upper positions. Some of these metabolites could play a role in nutrient remobilization.

## Introduction

Leaf senescence in crop plants influences agricultural yield through two different processes: photosynthesis and nutrient remobilization. As the major site of absorbing light energy and producing carbohydrates, chloroplasts get degraded when a leaf starts senescing. Life span of leaves or the duration of photosynthetic activities determines total assimilates available for grain filling thus crop yield^1^. Premature leaf senescence generally leads to significant yield loss while delayed senescence in mutants of many crop species showed increased grain production^2,3^. Senescence of leaves, on the other hand, is required for yield formation. During the process of leaf senescence, macromolecules such as proteins, lipids and polysaccharides are broken down into small mobile molecules which are transported through the vascular system to sink tissues including seeds^4–7^. It has been estimated that up to 95% of the proteins in cereal grains are synthesized from amino acids released from breaking down of existing proteins in leaves during senescence^8^.

Although the process of leaf senescence can be triggered by different signals such as phytohormones and environmental stresses, once the senescence program is initiated, as indicated by similar changes in gene expression, similar processes of senescence execution will be launched to achieve nutrient remobilization and ensure the completion of the life cycle of a plant^9,10^. The initiation and execution of leaf senescence are driven by differential expression of thousands of genes. Switches of gene expression are mediated by transcription factors. It has been shown that the major families of transcription factors associated with leaf senescence include NAC, WRKY, AP2⁄EREBP, MYB, C2H2 zinc-finger, bZIP, and GRAS^11–15^. A number of transcription factors, including the NAC family members NAP^16^, AtORE1/ANAC092^17^ and WRKY family member WRKY53^18^, have been shown to play an important role in regulating leaf senescence. Altered expression of these master regulators can lead to significant changes in the process of leaf senescence. Arabidopsis plants with AtNAP knocked-out, for example, displayed a phenotype of significantly delayed senescence while inducible overexpression of this gene caused precocious senescence^16^.

The execution processes of leaf senescence, including the degradation of macromolecules and remobilization of nutrients, are also controlled by differential gene expression. Metabolic enzymes including pyruvate orthophosphate dikinase (PPDK) and glutamine synthetase (GS) play key roles in facilitating nitrogen remobilization during leaf senescence. Overexpression of a gene encoding PPDK during senescence in Arabidopsis significantly accelerated nitrogen remobilization from leaves, leading to increased weight and nitrogen content of seeds^8^. Knockout of cytosolic GS genes reduced N remobilization as well as seed yield in Arabidopsis^19^.

During the past twenty years, transcriptomic as well as proteomic studies of leaf senescence have revealed a great deal of details on switches of primary and secondary metabolism during leaf senescence^9,20^. It has been shown that during leaf senescence, catabolism is up-regulated while anabolism is down-regulated^11^. Two recent articles reported studies on the metabolome of leaf senescence in Arabidopsis^21^ and sunflower^22^, respectively. Both studies revealed that during leaf senescence accumulation of the two amino acids that are believed to be the major forms of remobilized nitrogen for long distance transportation, glutamine and asparagine, increased. Increased accumulation of aromatic amino acids and branched amino acids was also observed in both studies. Changes of accumulation of sugars and TCA cycle products during leaf senescence, on the other hand, were more complex and seemed to be species dependent^21,22^.

A leaf can be a sink or source tissue at different developmental stages and/or at different positions of the plant. At late stages of development, nutrients remobilized in senescing leaves are transported to upper position sink tissues such as developing flowers, seeds, and young leaves. Recently a systems biological study on nutrient remobilization has been conducted by comparing the metabolomic data between sink and source tissues during leaf senescence^21^. Using the developing silique of Arabidopsis as a sink tissue, Watanabe *et al*. detected high accumulation of Met-derived-glucosinolates, flavonols and triacylglycerides in siliques in comparison with senescent leaves and suggested that these compounds might play a role in nutrient remobilization from senescing leaves to sink tissues^21^.

Tobacco (*Nicotiana tabacum*) is an economic crop and also an important model plant that has been widely used in plant biological research due to its high-efficient transformation and regeneration. Tobacco has also been used as a model system in studying leaf senescence^23,24^. One interesting feature of tobacco leaf senescence is that leaves of the same plant undergo senescence at a sequential manner: leaves at lower positions of the stalk senesce earlier such that along the main axis of a tobacco plant, a clear senescence gradation of the easily numbered leaves can be observed^23^. Among these leaves some are sink while some are source tissues, making tobacco a great model for studying nutrient remobilization from senescing leaves to sink tissues, in this case younger leaves at upper positions of the same plants.

In this study we used tobacco as a model system to study changes in transcriptome and metabolome during leaf senescence and the metabolic relationship between sink and source tissues. To this end, metabolomic and transcriptomic data from different leaf positions at different developmental stages were obtained and analyzed in combination. The results of this study unveiled a global picture of metabolic events related to nutrient remobilization during the progression of senescence.

## Results and Discussion

### Developmental leaf senescence in tobacco

**Figure 1:**
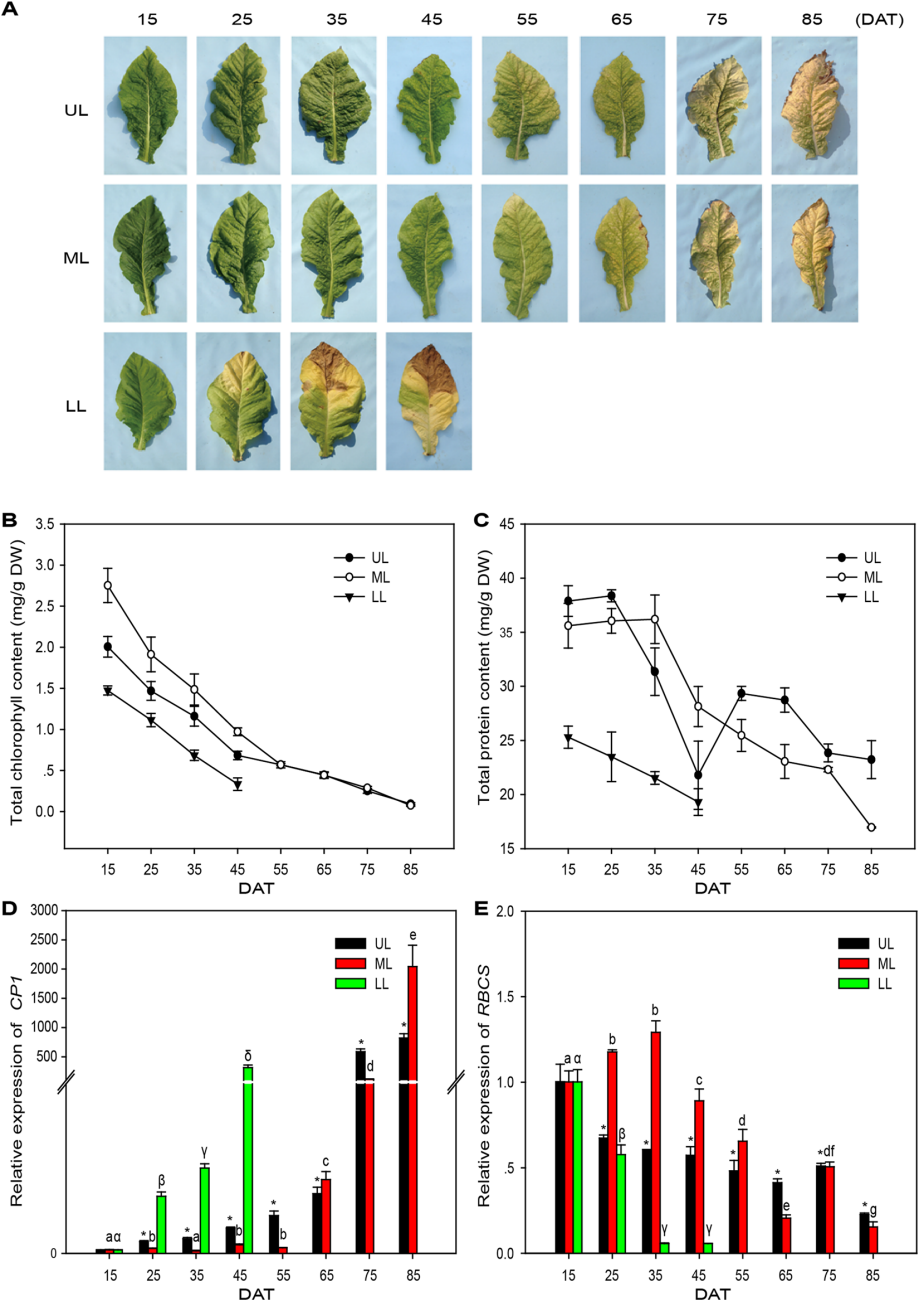
Developmental leaf senescence in tobacco. (**A**) Senescence stages of tobacco leaves used in this study. An example of upper leaf (UL), middle leaf (ML) and lower leaf (LL) harvested from plants from 15 to 85DAT. **(B**) and (**C**) Chlorophyll **(B)** and total protein **(C**) levels during leaf senescence. Data represent mean values of four biological replicates for each time point. The error bars indicate ± SD. DW, dry weight. **(D)** and (**E**) Transcript abundance of marker genes of leaf senescence. Expression levels of the genes were analyzed via qRT-PCR. Data are presented as mean ± SD. Values labelled with a different letter are statistically different from one another using one-way ANOVA test (p < 0.05). *Significant difference using student’s t-test (p < 0.05).

Similar to what has been described before^23^, tobacco leaves at different positions of a single plant undergo senescence in a sequential manner along the stalk, with leaves at lower positions undergo senescence first. To avoid effects of the topping practice, collections of leaf samples were not started until 15 days after topping (15DAT) and were conducted every 10 days thereafter. The sampling of leaves at a certain position was stopped when an average leaf at this position was completely senescent with desiccation. This resulted in leaf samples from eight time points (15 to 85DAT) at the upper and middle positions (UL and ML) and samples from four time points (15 to 45DAT) at the lower position (LL). The LL leaves started to show yellowing at the tip around 25DAT and was 50 to 90% yellow by 45DAT, while the UL and ML leaves remained green during this time course. After 45DAT, the UL and ML leaves displayed leaf yellowing over time in a manner similar to LL, with the margins and tips withered and dried out. By the final sampling time at 85DAT, all sampled leaves at UL and ML positions reached full senescence (Fig. 1A).

In agreement with the visible yellowing, chlorophyll contents and levels of soluble proteins in tobacco leaves decreased with the progression of development. The LL samples showed lower levels of chlorophyll and soluble proteins than the UL and ML leaves at the same sampling time (Fig. 1B, C). Furthermore, the expression of senescence marker genes was in line with the senescence phenotypes. The expression levels of *cysteine protease CP1* (SAG12 homolog in tobacco), which is normally used as a senescence marker gene, was up-regulated with the progression of leaf senescence. In LL, *CP1* up-regulation started at 25DAT (16.8- fold change against 15DAT) and the highest transcript abundance was observed at 45DAT, with a 313.9- fold change. In leaves at upper positions (UL and ML), however, transcripts of *CP1* did not show significant increase until at 45DAT (7.5- fold change in UL and 2.5- fold change in ML) and exhibited the highest expression levels at late stages of leaf senescence (821.8-fold change in UL and 1303.4- fold change in ML at 85DAT) (Fig. 1D). Consistent with the loss in chlorophyll, the transcript abundance of RBCS (ribulose bisphosphate carboxylase small subunit) encoding gene *LOC104241798* continuously decreased in LL from 15 to 45DAT, and showed a significant down regulation from 45 to 85DAT in UL/ML (Fig. 1E). The results from RNA-seq analysis showed the same expression patterns for these two marker genes as in qRT-PCR (Supplementary Fig. S1). Based on the changes of marker gene expression, LL started senescing from 15DAT while senescence of UL/ML started at 45DAT. It should be noted that probably due to environmental changes caused by weather conditions and/or agricultural practices around the sample harvesting time, there is an inconsistency in changes of senescence parameters (e.g., Fig. 1C) at 45DAT of UL.

### Profiling the transcriptome of tobacco leaf senescence

To understand the changes in gene expression in tobacco leaves at different leaf positions along the progression of developmental senescence, samples of UL, ML and LL leaves from each plant at 15, 25, 35, 45, 55, 65, 75, and 85DAT were collected and subjected to RNA-seq analysis (Fig. 1A). For each sample approximately 106 million clean reads with a mean length of 90 bp were obtained. The reads were assembled through *de novo* assembly to obtain 272,920 non-redundant unigenes (Supplementary Table S1). While the tobacco genome has been annotated to contain 69,595 genes^25^, the number of unigenes in this study was much bigger, potentially due to high redundancy of the unigene database from *de novo* transcriptome assembly. Based on gene family clustering, the 272,920 unigenes were divided into two classes: clusters and singletons. The former was prefixed with ‘CL’ and the latter with ‘unigene’. Each cluster contains multiple unigenes with high similarity (higher than 70%), which may be from the same gene or homologous genes. Each singleton, containing one unigene, represents one single gene. In total, 107,319 clusters and 145,601 singletons were obtained. Furthermore, high correlation coefficients (r = 0.891–0.997) were observed between the two biological replicates at all time points in RNA-seq data (Supplementary Fig. S2).

Approximately 75% of the unigenes (205,778) were annotated in the public databases, such as NR, SwissProt, COG, KEGG and GO (Supplementary Table S2 and Supplementary Fig. S3). Around 120,000 of the unigenes were classed into three gene ontology categories (cellular component, biological process and molecular function) with the cellular process (88,765 unigenes, 74%) and metabolic process (72,520 unigenes, 60.5%) category genes represented the major proportions. For the cellular component category, large numbers of unigenes were categorized as cell/cell part (88,765 unigenes), organelle (71,320 unigenes) and membrane (34,333 unigenes). For the molecular function category, catalytic activity (59,814 unigenes) and binding (56,577 unigenes) were the most abundant subcategories (Supplementary Fig. S4).

### Differentially expressed genes (DEGs) in tobacco leaf senescence

The significance of digital gene expression profiles was analyzed to identify DEGs^26^. For leaves from each position (UL, ML and LL), the expression levels of genes at the eight developmental stages were pair-wisely compared and hence 28 for UL, 28 for ML, and 6 comparisons for LL were performed respectively (Supplementary Table S3). To screen for genes differentially expressed during the senescence of tobacco, we set the first stage (15DAT) as a reference point and all later timepoints were compared with 15DAT. With the filter criteria of fold change >= 2.0 and false discovery rate (FDR) <= 0.001, 28,265 (10.4%), 43,059 (15.8%), and 42,547 (15.6%) DEGs were identified in UL, ML and LL leaves. For UL, 10,995 (38.9%) genes were only up-regulated, 9990 (35.3%) genes were only down-regulated, and 7280 genes were either up-down or down-up regulated. For ML, 17,371 (40.3%) genes were only up-regulated, 9450 (21.9%) genes were only down-regulated, and 16,238 genes showed either up-down or down-up regulated expression. For LL, 30,928 (72.7%) genes were only up-regulated, 7610 (17.9%) genes were only down-regulated, and 4009 genes were either up-down or down-up regulated. Greater number of senescence-associated genes (SAGs) in LL than UL and ML could be an indication of more nutrient remobilization activities in LL during senescence. As shown in Supplementary Fig. S5, the number of DEGs increased rapidly with leaf aging, suggesting that leaf senescence is not a passive and unregulated degeneration process.

**Figure 2:**
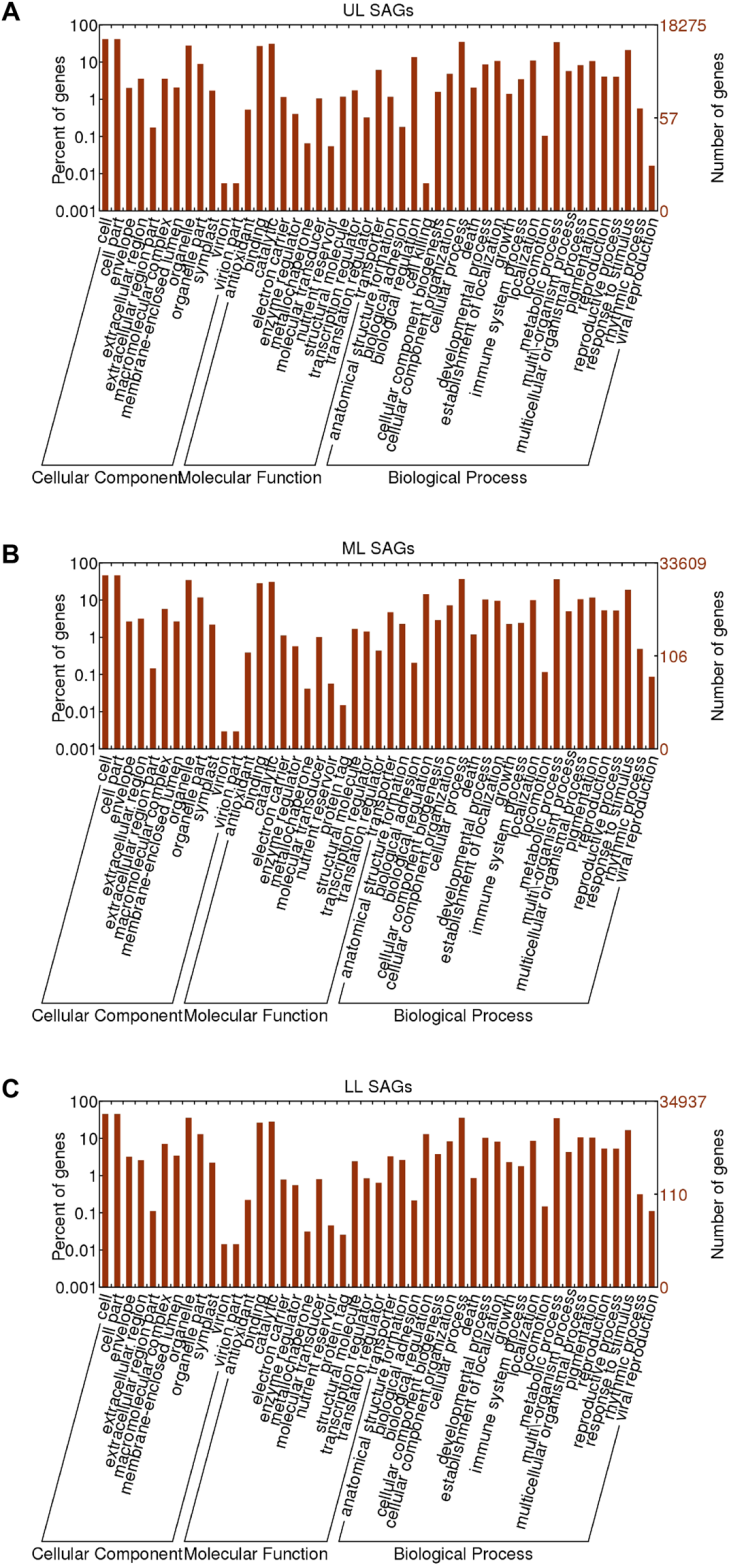
GO classification of SAGs in upper leaf (UL), middle leaf (ML) and lower leaf (LL). The X axis shows the three major categories analyzed. The Y axis shows the number of SAGs under each term.

Gene ontology (GO) analysis of the DEGs showed enrichment of SAGs in about 20 biological processes including biological regulation, developmental process, metabolic process, pigmentation and response to stimulus. In the molecular function aspect, SAGs were enriched in about 10 categories, including binding activity, catalytic activity, transporter and transcription regulator. In the cellular component aspect, SAGs were enriched in 12 categories including cell and cell part, organelle and organelle part, extracellular region and macromolecular complex (Fig. 2). Interestingly, similar GO categories were also enriched for the senescence-down-regulated genes (SDGs) (Supplementary Fig. S6). In GO categories metabolic processes and catalytic activity, the numbers of SAGs were significantly larger than those of SDGs, suggesting an enhancement of catalytic activities during leaf senescence. SAGs related to protein tagging activities were enriched in ML and LL but not in UL leaves. Protein tagging could be an indication of degradation and remobilization - when a molecule stops functioning or is no longer needed, it might be attached by special tags which usually results in modification, sequestration, transport and/or degradation.

Pathway enrichment analysis showed that tobacco SAGs were enriched in 35, 42, 42 pathways in UL, ML, and LL, respectively (Q-value <= 0.05) (Supplementary Table S4). The enriched pathways included carbohydrate metabolism such as starch, sucrose and galactose metabolism, amino sugar and nucleotide sugar metabolism, ascorbate and aldarate metabolism pathways. Pathways related to biosynthesis of secondary metabolites were also overrepresented. These included pathways of phenylpropanoid biosynthesis, stilbenoid, diarylheptanoid and gingerol biosynthesis, flavonoid biosynthesis, flavone and flavonol biosynthesis. Pathways related to amino acid metabolism such as lysine degradation, arginine and proline metabolism, alanine, aspartate and glutamate metabolism were also overrepresented. Interestingly, the most abundant transcripts in LL were related to genetic information processing and cellular processes such as ribosome (Ko03010, 876 genes, 6.96%), spliceosome (Ko03040, 794 genes, 6.31%), RNA transport (Ko03013, 785 genes, 6.24%), protein processing in endoplasmic reticulum (Ko04141, 655 genes, 5.2%), phagosome (Ko04145, 279 genes, 2.21%) and peroxisome (Ko04146, 244 genes, 1.94%).

To provide a global view of tobacco leaf metabolism, DEGs from different leaf positions (UL, ML and LL) with different KO ids were submitted for analysis via the on-line Interactive Pathways (ipath) explorer v2 and mapped to metabolism pathways. The results showed that while the expression of genes in many metabolic pathways decreased significantly during leaf senescence, a big number of pathways, including those involved in carbohydrate metabolism, energy metabolism and amino acid metabolism, showed enhancement during senescence (Supplementary Fig. S7). An overall up-regulation of the gene expression machinery including ribosomal proteins and RNA polymerases (data not shown), further supported the idea that leaf senescence is an exceedingly active process–with metabolism of specific set of metabolic pathways more induced in old leaves than young one^27^.

Comparison of the results from KEGG pathway enrichment analysis and interactive pathways (Ipath) analysis indicated that some pathways and genes showed distinctive expression patterns between leaves from different positions. For example, during senescence carotenoid biosynthesis and biosynthesis of aromatic amino acids such as phenylalanine, tyrosine and tryptophan were enhanced in ML and LL but decreased in UL. Genes encoding for ABC transporters were up-regulated in UL but down-regulated in ML and LL. The difference between different leaf positions in expression changes of these enzyme-encoding genes may reflect a sink-source transition. More catalytic activities in lower position/source leaves and more transportation activities in upper position/sink leaves are indications of sink/source identities as well as the direction of nutrient remobilization.

### The metabolome of tobacco leaf senescence

In order to investigate the metabolic kinetics of tobacco leaves at different leaf positions along the progression of developmental senescence, samples of UL, ML and LL leaves at 15, 25, 35, 45, 55, 65, 75, and 85DAT were collected and subjected to metabolic analysis using gas chromatography (GC)-mass spectrometry (MS) and ultra-high performance liquid chromatography (UPLC). Totally seventy six metabolites were identified and quantified, including 15 carbohydrates, 22 amino acids, 19 organic acids and lipids, 3 nucleotides, 15 secondary metabolites (including polyphenols, alkaloids and terpenoids) and two other compounds (Supplementary Table S5 and Supplementary Table S6). The identities of 39 out of the 76 metabolites were further verified using reference standards.

**Figure 3:**
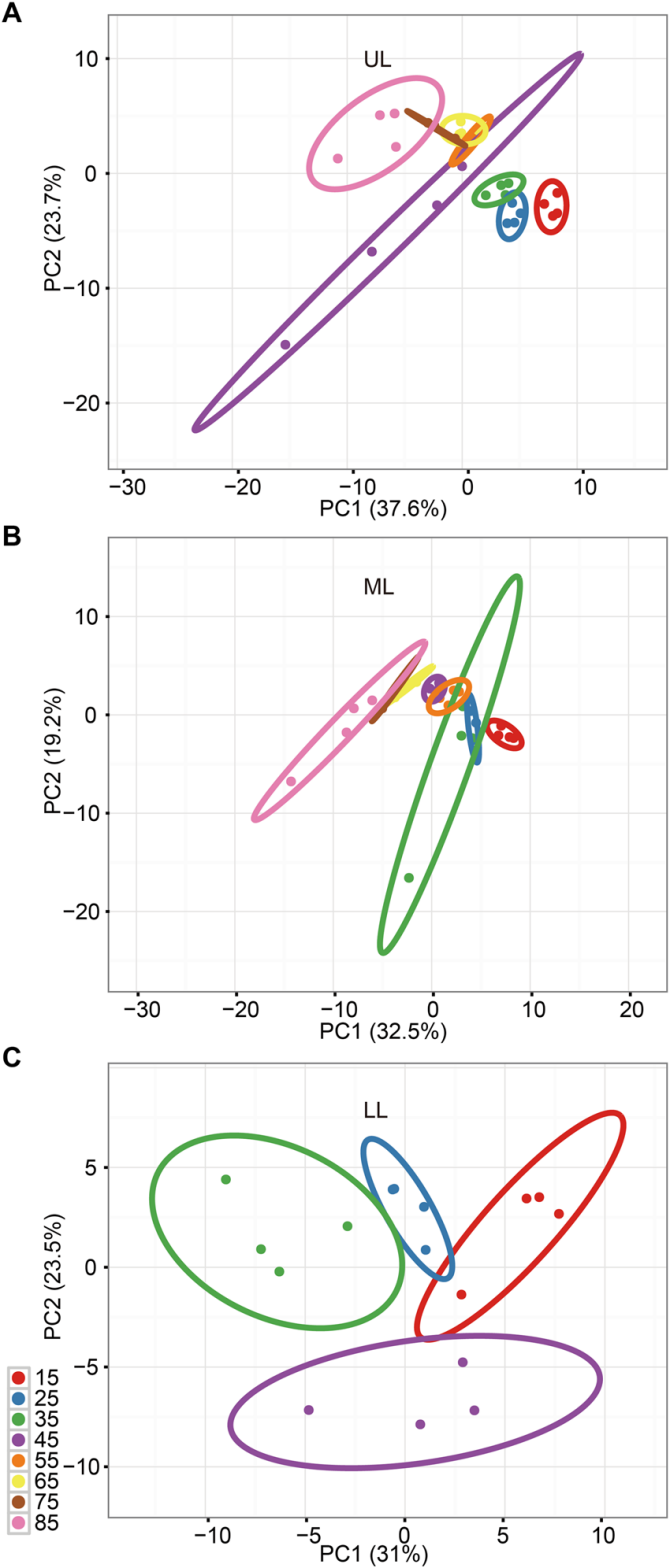
Principal component analysis (PCA) of metabolite profiles from **(A)** upper leaf, **(B)** middle leaf and **(C)** Lower leaf during tobacco leaf senescence. Each point represents an independent biological replicate. Red, blue, green, purple, orange, yellow, brown and pink colors represent samples at 15, 25, 35, 45, 55, 65, 75 and 85DAT, respectively. Triangle, box and dot denote metabolomes of UL, ML and LL, respectively. Plotting of the first and second component is shown. The circles indicate the 95% confident regions.

Significant changes in metabolite accumulation were observed between leaf samples from different developmental stages and from different leaf positions. All of the 76 primary and secondary metabolites were subjected to Principal Component Analysis (PCA) to visualize the general trend of metabolic changes in respect to developmental stages. Notably, PCA of metabolite data showed a lower variability among biological replicates. Leaves from the same position showed an evident difference between different developmental stages, indicating significant changes in primary and secondary metabolism during leaf senescence (Fig. 3A–C and Supplementary Fig. S8). Meanwhile, the PCA analysis also revealed dynamic patterns of changes in metabolism between upper and lower position leaves (UL and ML versus LL). For the upper position leaves (UL and ML), the metabolic changes from 15 to 85DAT showed a linear trend, while for the LL leaves a scattered distribution of principal components was observed with no apparent trend with the progression of senescence. Different patterns of metabolic changes between leaf positions might reflect sink-source transitions.

### Transcriptional and metabolic analyses reveal substantial changes in primary and secondary metabolites during leaf senescence

#### Sugars

Sugars have been suggested to regulate plant growth, photosynthetic rate and the developmental progression of leaf senescence^28–32^. However, the role of sugars in plant senescence remains controversial and differs between different species^30–34^. Increased accumulation of sugars during leaf development has been reported in Arabidopsis and tobacco^21,23,35,36^. The results of sugar accumulation during sunflower leaf senescence, however, have been contradictory^22,37,38^. Both sugar addition and starvation can induce SAG expression and early senescence^32,39,40^. The role of sugars in the senescence program may vary in different species, treatments and tissue types^34^.

**Figure 4:**
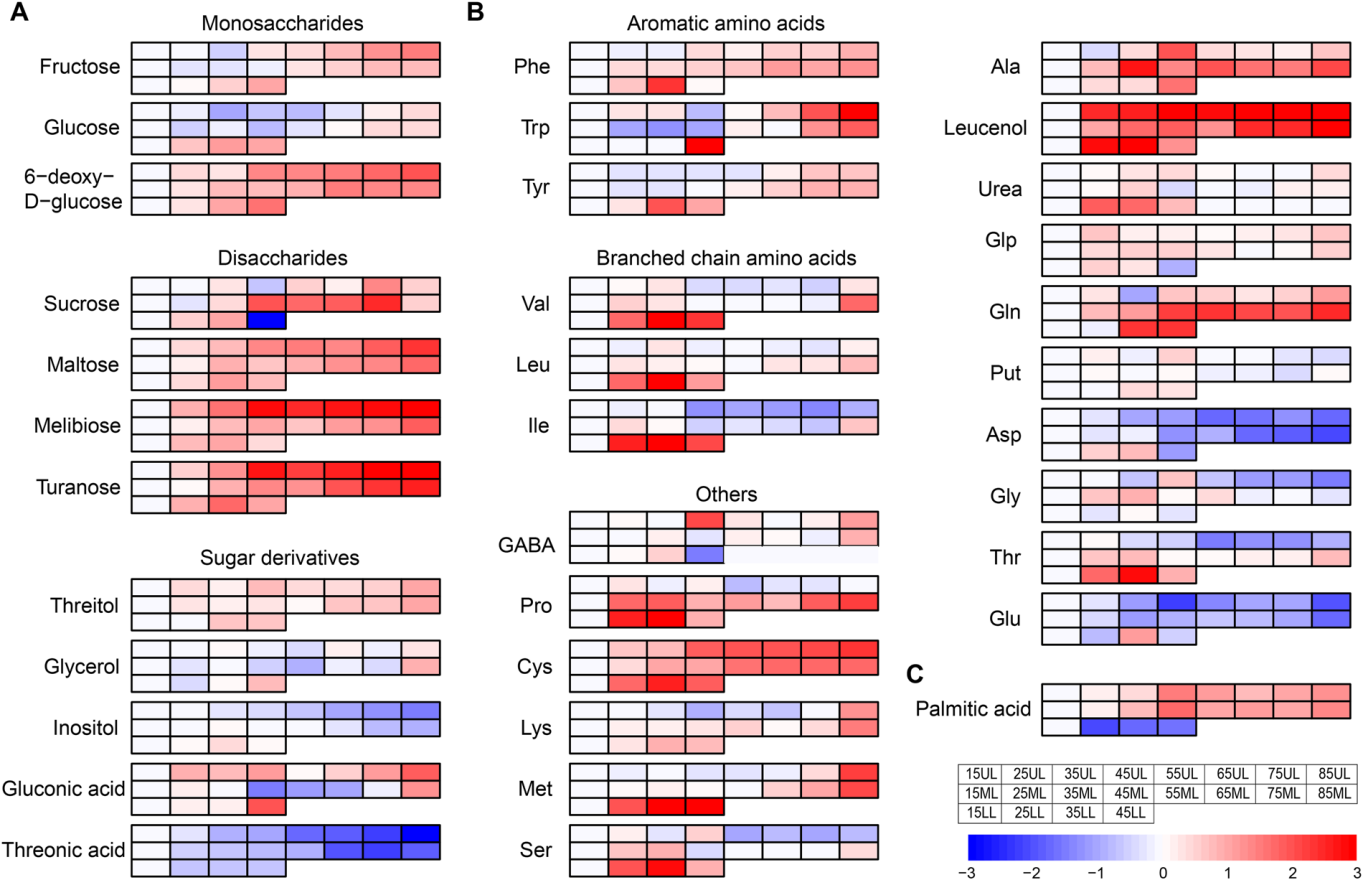
Heat map of metabolite changes during leaf senescence. Log2 ratios of fold changes from 15DAT are given by shades of red (up-regulation) or blue (down-regulation) colors as indicated in the scale bar. Data represent mean values of four biological replicates for each leaf position and time point. **(A)** Sugars. **(B)** Amino acids. **(C)** Fatty acid.

In this study, for tobacco leaves from all three stalk positions, as a general trend, metabolites associated with sugar metabolism increased as the leaf yellowing. This included monosaccharides fructose, glucose and 6-deoxy-D-glucose and disaccharides sucrose, maltose, melibiose and turanose. Alongside the increase of sugars during leaf senescence, accumulation of sugar derivatives such as sugar alcohols (threitol, glycerol) and aldonic acids (gluconic acid) also increased, while the accumulation of inositol (sugar alcohol) and threonic acid (sugar acid) decreased during senescence (Fig. 4A). Of particular interest, sugars (such as glucose, fructose) from the UL position showed a biphasic pattern of change, decreased first from 15 to 35DAT and started to increase significantly at 45DAT. The decline in sugar contents during the first 30 days (when the UL leaves were still expanding) may be due to the continuous use of carbohydrate resources produced via photosynthesis for cell elongation and division. Sugars (sucrose, maltose, melibiose and turanose) in the LL, on the other hand, decreased at the late stage of senescence (45DAT) when sugar contents in the UL or/and ML started to increase. The concurrence of sugar reduction at lower positions and sugar increase at upper positions may be an indication of sink/source transitions.

**Figure 5:**
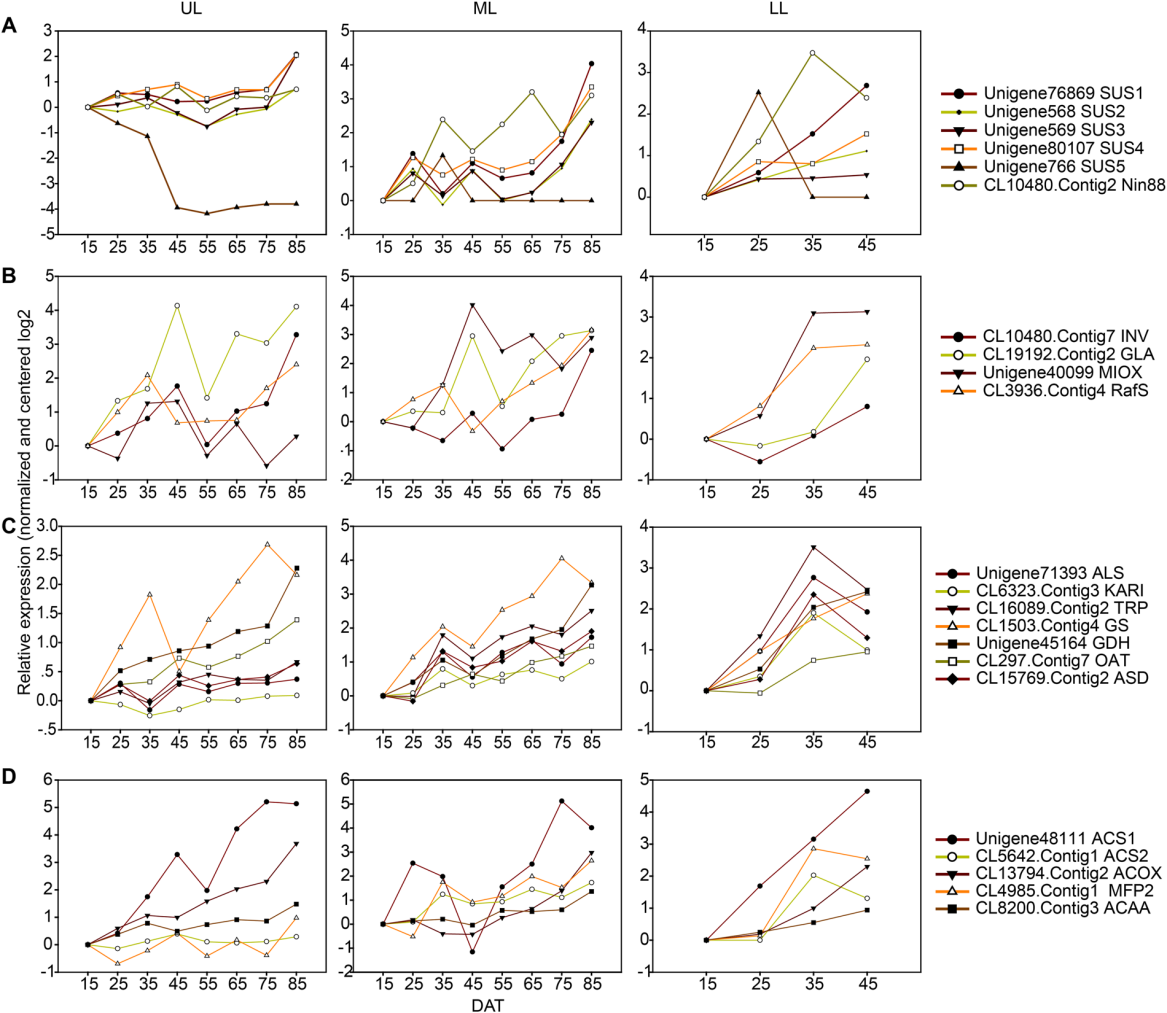
Expression patterns of transcripts involved in sugar, amino acid and fatty acid metabolism at different leaf positions during senescence. Relative expression levels of transcripts selected from the RNA-seq data (means of two biological replicates, log2 transformed fold changes normalized to 15DAT of each part leaf). The X axis refers to each stage of leaf development. The Y axis represents gene expression levels. **(A**) Sugar metabolism. **(B)** Metabolism of minor sugars (galactinol, melibiose and inositol). **(C)** Amino acid metabolism. (**D**) Fatty acid metabolism.

Consistent with the metabolomic data, the RNA-seq analysis indicated that the expression of genes related to the sugar metabolic pathways was up-regulated during senescence. Among them, two important enzyme families, invertases (INVs) and sucrose synthases (SUSs), showed high expression in senescent leaves in tobacco. A gene encoding extracellular invertase Nin88 (*CL10480.Contig2*) that facilitates sucrose degradation was ~10- fold up-regulated in LL from 15 to 45DAT. Expression of the same gene was also significantly increased in ML (8.56- fold change) and UL (1.63- fold change) at 85DAT (Fig. 5A). This gene has been shown to be involved in sugar partitioning as the key enzyme of an apoplastic phloem unloading pathway and function in source-sink regulation^41^. Moreover, four transcripts coding for SUSs, *Unigene76869*, *Unigene568*, *Unigene569* and *Unigene80107*, were highly induced following senescence while the other sucrose synthase gene, *Unigene766*, was down-regulated in UL from 45 to 85DAT (Fig. 5A). SUS enzymes catalyze the reversible conversion of sucrose and UDP into fructose and UDP-glucose, that are widely considered as the key enzyme involved in the plant sugar metabolism. Previous studies have shown that they have important roles in a number of important metabolic processes, such as sucrose distribution between plant source and sink organs^42,43^, nitrogen fixation^44^, starch and cellulose synthesis^45^ and environmental stress responses^43,46^. Altogether, these results suggest high sucrose mobility throughout the leaf senescence process in tobacco plants and both types of sucrose metabolic enzymes might be actively involved in remobilization of sucrose from senescing leaves into sink tissues. It has been suggested that in addition to their catalytic activities in sucrose metabolism, SUSs could play a role in inter-compartmental signaling^47,48^. It will be interesting to see whether some of the senescence-associated tobacco SUSs might play a role in regulating leaf senescence.

A number of genes involved in the metabolism of minor carbohydrates (minor sugars) were also induced during leaf senescence, including genes encoding inositol oxygenase (MIOX) (*Unigene40099*), beta-fructofuranosidase (INV) (*CL10480.Contig7*), alpha-galactosidase (GLA) (*CL19192.Contig2*) and raffinose synthase (RafS) (*CL3936.Contig4*) (Fig. 5B). For example, one transcript coding for MIOX (*Unigene40099*) increased significantly in ML (7.4- fold change) and LL (8.8- fold change) at 85DAT. The enzyme inositol oxygenase catalyzes the oxidation of inositol into glucuronic acid. The decreased level of inositol in old leaves could be the consequence of highly inducible gene expression of inositol oxygenase-encoding genes during leaf senescence (Fig. 4A).

#### Amino acids

The concentrations 22 proteinogenic and nonproteinogeinc amino acid were determined in this study (Fig. 4B). The accumulation of aromatic amino acids, which are derivatives of the shikimate pathway, including Phe, Trp and Tyr, were observed to have significant increase during leaf senescence. This class of amino acids have been previously reported to accumulate during developmental and nitrate limitation/dark-induced senescence^21,36,49–51^. Aromatic amino acids are important precursors for the synthesis of secondary metabolites such as flavonoids. Flavonoids have been shown to act as cellular protectants during senescence^52^. Branched chain amino acids Val, Leu and Ile, which have been shown to be critical in respiration as alternative substrates under dark-induced senescence^49,50^, also displayed high level accumulation throughout leaf senescence, especially in LL. A similar pattern of increase was also observed for Pro. Pro is often documented to be a stress-induced osmoprotectant and its accumulation has been reported to be associated with leaf senescence^21,35,53–55^. In addition, significantly increased accumulation during senescence was also observed for Lys, Cys, Gln, Met, as well as Glu-derived pyro-Glu (Glp) and Ala-derived Leucenol. Gly, Glu and Asp, on the other hand were significantly reduced with the progression of senescence. It is notable that the nitrogen-rich amino acid Gln accumulated concomitantly with the decrease of Glu when the leaves aged, suggesting mobilization and reallocation of nitrogen from senescing leaves to sink leaves (Fig. 4B).

Protein degradation plays a critical role in releasing free amino acids for nutrient recycling during senescence. Protease-encoding genes show highly induced expression during leaf senescence in different species^22,56–58^. In the present study, genes associated with protein processing and proteolysis were also transcriptonally up-regulated during senescence. This includes subtilisin-like proteases and cysteine proteases, including a homologue to Arabidopsis SAG12 (tobacco *cysteine protease CP1*), which is specifically expressed in senescent tissues^59^.

Another source of amino acids is biosynthesis. Several amino acid biosynthesis genes were up-regulated during tobacco leaf senescence in our study. For instance, in agreement with the high accumulation of branched amino acids in senescing leaves, most genes in the valine, leucine and isoleucine biosynthesis pathway were up-regulated as leaf yellowing. In particular, expression of the genes coding for the first two step enzymes, acetolactate synthase (ALS) and ketol-acid reductoisomerase (KARI), were significantly up-regulated in LL, potentially contributed to the high accumulation of branched chain amino acids in LL. The genes coding for tryptophan synthase (TRP), aspartate-semialdehyde dehydrogenase (ASD), glutamine synthetase (GS) and glutamate dehydrogenase (GDH) were also significantly up-regulated, supporting the substantially increased accumulation of their catalytic products Trp, Lys and Gln in senescing leaves. Similarly, consistent with the senescence-associated accumulation of Pro, the ornithine aminotransferase-encoding gene (OAT) was continuously up-regulated from 15 to 85DAT (Fig. 5C). Ornithine aminotransferase catalyzes the ultimate formation of the Pro from ornithine.

#### Fatty acids and beta-oxidation

An enhancement of fatty acid catabolism was observed during tobacco leaf senescence. The level of saturated fatty acid palmitic acid showed a mixed pattern at different leaf positions. In LL, palmitate was sharply decreased from 15 to 25DAT and remained constant until 45DAT. Whereas in the UL and ML, accumulation of palmitate increased significantly at 45DAT and slightly decreased after that (Fig. 4C). Meanwhile expression of genes in fatty acid catabolism, beta-oxidation in particular, such as acyl-CoA synthetase (ACS1/2), acyl-CoA oxidase (ACOX), enoyl-CoA hydratase/3-hydroxyacyl-CoA dehydrogenase (MFP2) and acetyl-CoA acyltransferase (ACAA) was in accordance with the changes in metabolite contents. In LL, a significant up-regulation of most of the above-mentioned beta-oxidation genes was observed while UL and ML showed an up-regulation of the same genes only after 45DAT (Fig. 5D). The beta-oxidation process leads to the production of acetyl-CoA, the substrate of the tricarboxylic acid (TCA) cycle.

#### The TCA cycle

**Figure 6:**
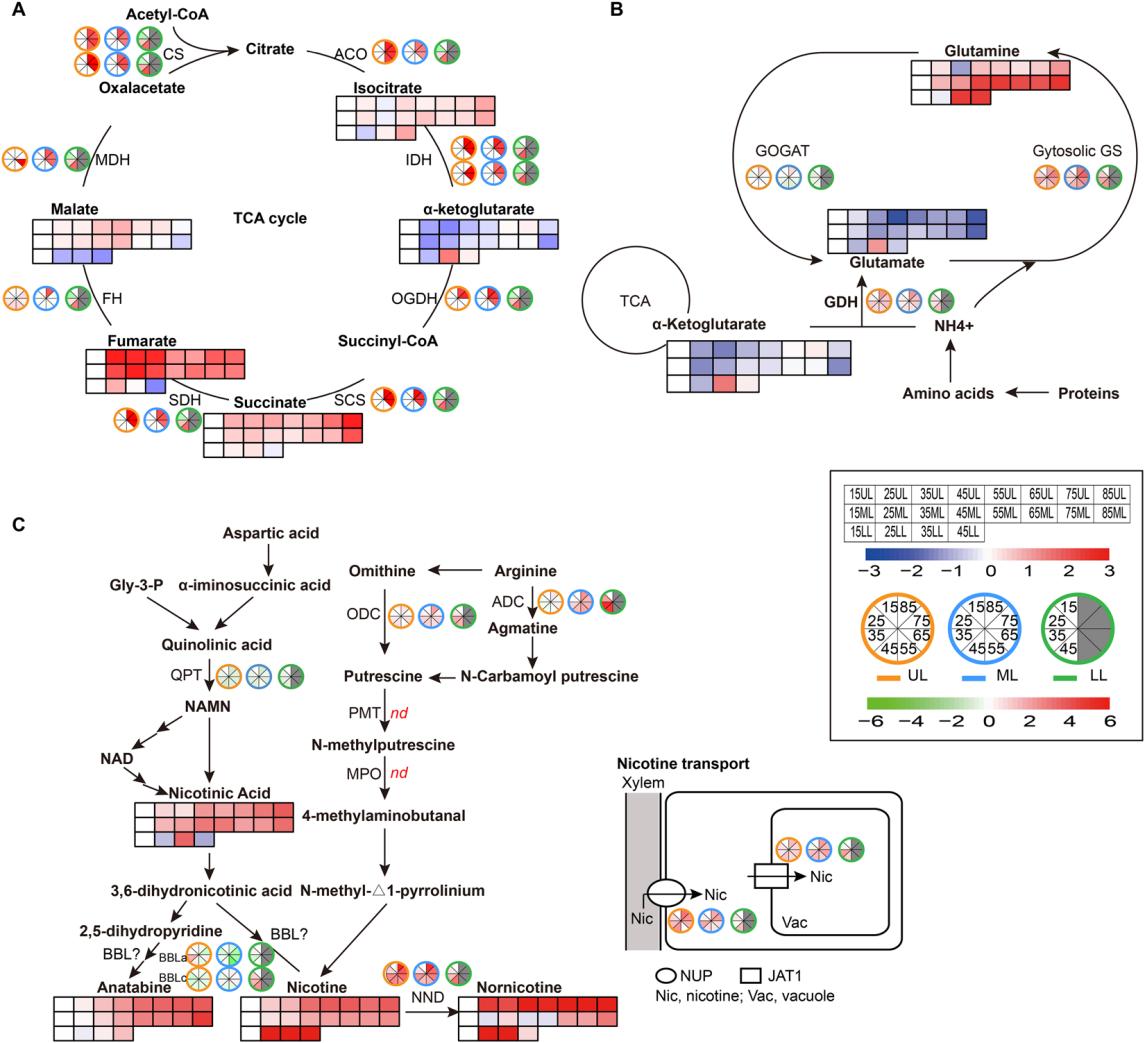
Heat map of changes in metabolites and transcripts in the **(A)** TCA cycle, **(B)** nitrogen recycling and **(C)** nicotine metabolism during leaf senescence. Metabolites and transcripts are represented by squares and circles, respectively. Log2 ratios of fold changes from 15DAT are given by shades of red (up-regulation) or blue (down-regulation of metabolites) and green (down-regulation of genes) colors as indicated in the scale bar. Multiple transcripts matching each gene are shown side by side near the gene names. Abbreviation were defined as follows: CS, citrate synthase. ACO, aconitase. IDH, isocitrate dehydrogenase. OGDH, alfa-ketoglutarate dehydrogenase. SCS, succinyl-CoA synthetase. SDH, succinate dehydrogenase. FH, fumarate hydratase. MDH, malate dehydrogenase. Cytosolic GS, cytosolic glutamine synthetase. GOGAT, NADH-dependent glutamate synthase. GDH, glutamate dehydrogenase. ADC, arginine decarboxylase. ODC, omithine decatboxylase. QPT, quinolinate phosphoribosyltransferase. NND, nicotine N-demethylase. BBLa/BBLc, berberine bridge enzyme-like family gene. JAT1, jasmonate-inducible alkaloid transporter1. NUP, nicotine uptake permease.

Despite the constant increase in sugars during senescence, the TCA cycle intermediates displayed a rather mixed pattern. As shown in Fig. 6A, the levels of isocitrate, α-ketoglutarate and succinate increased during leaf senescence, while the change of malate and fumarate showed a biphasic pattern, increased at the early stages then declined during the later stages. At the transcriptional level, genes encoding for TCA cycle enzymes such as citrate synthase (CS), aconitase (ACO), isocitrate dehydrogenase (IDH), α-ketoglutarate dehydrogenase (OGDH), succinyl-CoA synthetase (SCS), succinate dehydrogenase (SDH), fumarate hydratase (FH) and malate dehydrogenase (MDH) were up-regulated following leaf senescence. Among them, the three rate-limiting enzyme genes (CS, IDH and OGDH) were induced significantly as the leaf yellowing. For example, at the last stage (85DAT for UL and ML, 45DAT for LL), two CS coding genes (*Unigene65108* and *Unigene41903*) showed obvious up-regulation (93.1- and 22.2- fold changes in UL; 13.5- and 18.2- fold changes in ML; 14.9- and 10.8- fold changes in LL, respectively). Two transcripts coding for IDH genes (*Unigene2102* and *Unigene1484*) were also induced significantly (8.4- and 8.9- fold changes in UL; 5.1- and 3.6- fold changes in ML; 3.6- and 3.7- fold changes in LL, respectively). In addition, one transcript (*Unigene51974*) coding for OGDH genes showed significant up-regulation at every leaf position at the last stage (23.6- fold change in LL, 36.6- fold change in ML and 12- fold change in LL, respectively). The results from both metabolomic and transcriptomic analyses suggested increased activities of the TCA cycle during senescence, which is consistent with the results of a previous proteomic study showing that the proteins involved in TCA cycle were induced during leaf senescence^60^. The TCA cycle functions in the mitochondrion to provide intermediates for generating ATP. These results support the hypothesis that mitochondria remain functional until very late stages of leaf senescence^61^. The progressive rise of TCA cycle activities likely matches the increased demand of energy for cell viability maintenance and nutrient mobilization in senescing leaves.

#### Nitrogen recycling

As a leaf senesces, nutrients such as nitrogen, phosphorus, and minerals are remobilized to rapidly growing and/or storage organs of the plant. It has been estimated that up to 90% of the nitrogen in a senescing leaf is recycled^62–64^. In the senescing leaves, a large amount of ammonium is produced as a result of protein and nucleic acid catabolism^65,66^. The ammonium group could be channeled back into amino acid structures via the GS/GOGAT cycle which is carried out by the concerted action of glutamine synthetase (GS) and glutamate synthase (GOGAT)^67,68^. In addition, ammonium could also be directly incorporated into glutamate through amination of α-Ketoglutarate by the mitochondrial glutamate dehydrogenase (NADH-GDH) and subsequently into glutamine by cytosolic GS^69,70^. In this study, the levels of transcripts of cytosolic GS and GDH showed tight association with leaf senescence (Fig. 6B). During the leaf aging, cytosolic GS (GS1) (*CL1503.Contig4*) was highly up-regulated in leaves at all three positions, while chloroplastic GS (GS2) was down-regulated, potentially as the result of chloroplast collapse. In addition, GDH (*CL267.Contig14*) was also up-regulated during the aging process of tobacco leaves. The induction of GS and GDH during senescence has been reported in Arabidopsis and tobacco before^11,23,71^. In contrast, GOGAT (*CL9743.Contig3*), the other enzyme in the GS/GOGAT cycle, which catalyzes the conversion of glutamine to refill the glutamate pool, was down-regulated (0.74- fold change) in LL at 45DAT and in UL/ML during early stages of senescence. All this suggests that nitrogen in senescing leaves of tobacco is accumulating in the form of glutamine, which might be the major form of nitrogen recycling.

In agreement with the transcriptomic data, glutamine showed high levels during tobacco leaf senescence. Glutamate on the other hand, showed a biphasic pattern of change, increased transiently at early senescing stages but decreased at the late stages. This suggests that these amino acids, which are rich in nitrogen, could be efficiently inter-converted and exported from the senescing leaves (Fig. 6B). The decrease of α-Ketoglutarate in our study also suggests GDH might function in the aminating direction and is important in nitrogen assimilation and recycling. For all parts of tobacco leaves, glutamine, as the major phloem-exported amino acid for long distance transport of N, was accumulated as leaf yellowing in leaves at all three positions (UL, ML and LL).

Overall, during leaf senescence, the GS/GOGAT cycle seemed to work towards production of more glutamine, which might be further transported to sink tissues through long distance transportation. The results of a recent study on tobacco leaf metabolome at early senescence stages, however, suggested a continuous decrease in glutamine content in middle leaves from the full bloom stage (S3) to the middle leaf ripening stage (S5)^35^. Tobacco plants were topped at the S3 stage and lower leaves were harvested at the S4 stage in the above-mentioned study. The practices of topping and harvesting could cause sink-source switches and have significant effects on metabolism in leaves of the same plant. In the current study, in order to avoid the influence of agricultural practices, sample harvest was not started until 15 days after topping and no leaf was harvested in the plants for sampling (plants used for sampling at an earlier time point was no longer used for later samplings).

#### Nicotine metabolism

Besides primary metabolites, GC-MS and UPLC based metabolic profiling also revealed major changes in some secondary metabolites. In this study, 15 secondary metabolites were identified and measured during different stages of senescence. Among them, significant increase in levels of eight phenolics and four alkaloids were observed during senescence. Enhanced accumulation of secondary metabolites and up-regulation of related biosynthetic pathways, many of which are defense-related, have been previously observed during senescence^11,21^. High accumulation of certain secondary metabolites may play a protective role in ensuring viability of senescing cells to facilitate nutrient recycling. As will be discussed below, some of these metabolites may also be transported to sink tissues.

As a unique group of secondary metabolites, alkaloids are of special interest for tobacco growers. The major alkaloid nicotine has been implicated in plant antiherbivore defense and smoking addiction^72,73^. In a typical commercial tobacco plant, nicotine comprises about 90% of the total alkaloid pool, with the alkaloids nornicotine (a demethylated derivative of nicotine), anatabine and anabasine making up most of the remainder. The results from this study indicated that alkaloid/nicotine metabolism was enhanced during leaf senescence. The content of alkaloids nicotine, nornicotine, anatabine, as well as the nicotine biosynthesis intermediate nicotinic acid, all increased constantly from 15 to 45/85DAT at all leaf positions (Fig. 6C). Among the nicotine biosynthesis genes, transcripts coding for arginine decarboxylase (ADC, *CL5593.Contig8*) and omithine decatboxylase (ODC, *Unigene35722*) were highly up-regulated during senescence at all leaf positions. However, the quinolinate phosphoribosyltransferase-encoding gene (QPT, *CL3438.Contig1*) was down-regulated, indicating differential expression between the methylpyrroline pathway and pyridine-nucleotide cyclic pathway. In addition, transcripts coding for two berberine bridge enzyme (BBLa, *CL517.Contig1* and BBLc, *CL517.Contig2*) family members, which are involved in a late, possibly the final, step of tobacco alkaloid biosynthesis, were down-regulated during senescence. Previous studies showed that nicotine is synthesized exclusively in roots, then translocated to leaves via long distance transportation, and finally accumulated in the leaf vacuoles^74,75^. Hence, the continuous accumulation of nicotine in leaves from 15 to 85DAT may be due to the long-distance transport from roots or lower position leaves. In agreement with this, the expression of two alkaloid transporter, nicotine uptake permease (NUP) (*Unigene46090*) and vacuolar-type jasmonate-inducible alkaloid transporter1 (JAT1) (*CL1529.Contig6*) were also shown to be up-regulated. The nice association between metabolites and related transcripts in nicotine metabolism observed in this study, however, raises the question whether alkaloid biosynthesis also exist in leaves or not. The nicotine N-demethylase-coding (NND) transcript (*Unigene109139*) was induced significantly as leaf aging, supporting the substantial accumulation of nornicotine during leaf senescence (Fig. 6C).

#### Metabolites for long-distance transportation during leaf senescence

Leaf senescence is an important step in the life cycle of annual plants as it allows for mobilization and recycling of the nutrients to developing seeds to prepare for the next generation. Furthermore, nutrient remobilization from senescing leaves to developing organs during plant senescence is critical for plant yield and quality^4,76,77^. Once the senescence program is initiated, the cellular biochemical changes in senescing leaves are accompanied by reduced anabolism and enhanced catabolism, with macromolecules like proteins, RNA, and membrane lipids being degraded and released nutrients recycled. The translocation of phloem-mobile nutrients such as sugars, amino acids and nicotine during the sink-source transition has been previously reported^67,78–80^.

**Figure 7:**
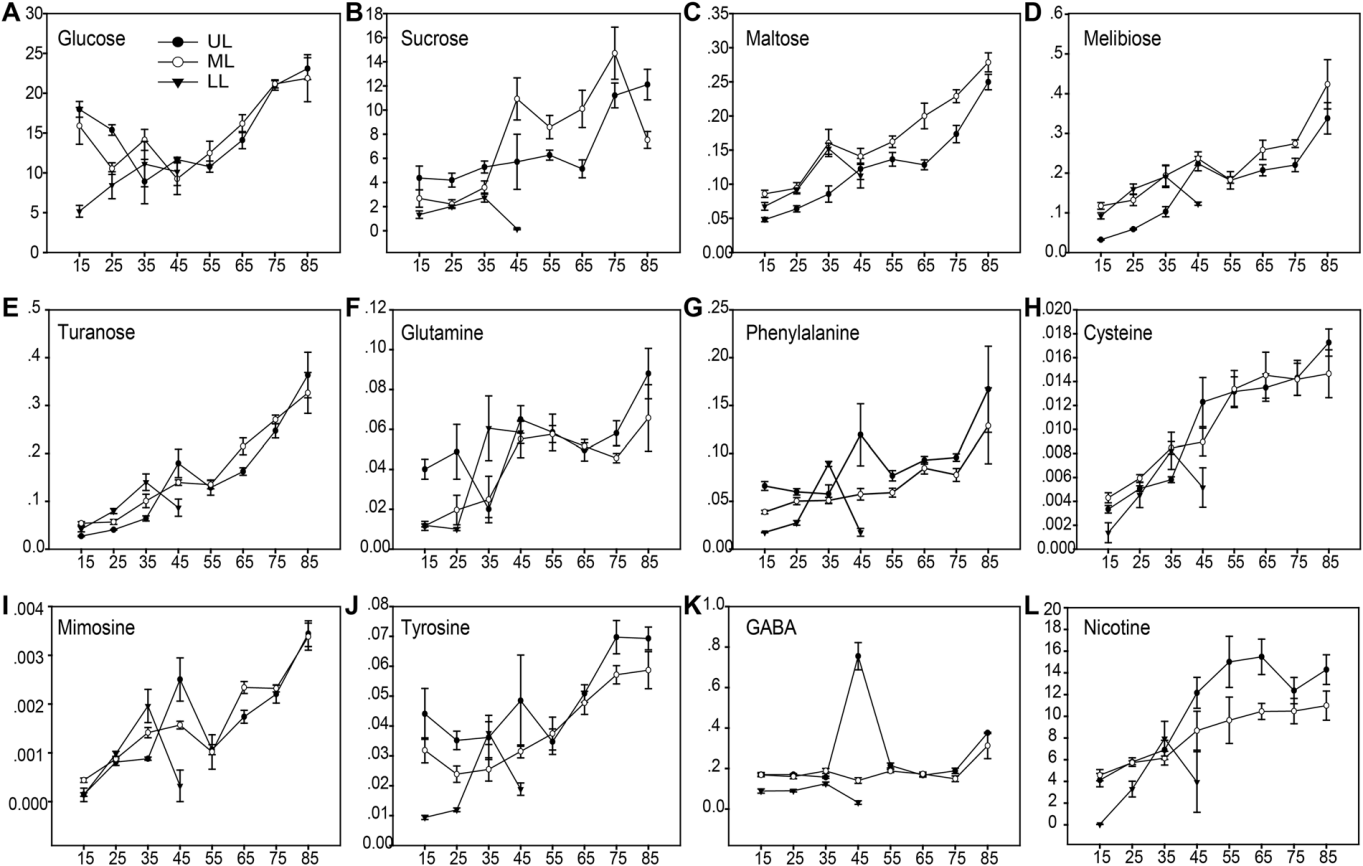
Changes of the selected metabolites which are candidate metabolites for long distance transportation during senescence.

In the current study, tobacco plants with inflorescence removed were used to study nutrient remobilization during leaf senescence. In this system, lower position leaves senesce earlier such that leaves at different stalk positions function as source and sink tissues respectively. Changes of metabolite contents during senescence within different position leaves were determined to investigate the changes of metabolism caused by sink-source transition and nutrient flux between leaves at different positions/senescence stages. A comparison of the levels of the metabolites between leaf positions revealed specific distribution patterns with the progression of senescence. Most of the sugars (glucose, sucrose, maltose, melibiose, turanose) showed much higher accumulation in upper parts of the plant at late stages of senescence (Fig. 7A–E). Interestingly, the time of the sugars started increasing in upper leaves coincided with their decrease in lower leaves. For example, the sucrose content increased in LL but stayed constant in UL and ML before 35DAT. After 35DAT, accumulation of sucrose started to increase in UL and ML while decreased sharply in LL leaves, suggesting active export of sucrose from lower to the upper position leaves (Fig. 7B). Two transcripts encoding for the sucrose transporter SUT, which plays an important role in mediating sucrose partitioning in plants^81–84^, were detected in this study. Both SUT encoding genes, *CL10296.Contig1* and *CL1413.Contig12*, showed significant increase in expression after 35DAT, although the former was somewhat down-regulated at late stages of senescence in ML and UL (Supplementary Fig. S10A,B). The up-regulation of SUT-encoding genes from 35 to 45DAT seemed to be in accordance with the transport of sucrose from senescing to sink leaves. Furthermore, two transcripts coding for UDP-galactose/UDP-glucose transporters (UTR) (*Unigene21801* and *Unigene72544*) were up-regulated with aging in LL, while down-regulated in UL and ML (Supplementary Fig. S10C,D). Overall, the results of metabolomic and trancriptomic analyses in sugar metabolism suggested active transportation of phloem-mobile sugars from senescing to sink leaves. Similarly, the glutamine, which have been shown to be accumulated as leaf senescing in the “Nitrogen recycling” part, also displayed active transportation from senescing leaves to sink leaves (Fig. 7F). In addition, the levels of several other amino acids, including phenylalanine, cysteine, mimosine, tyrosine and GABA, also showed the similar pattern of changes at different leaf positions during senescence, suggesting active amino acids remobilization throughout the leaf senescence in tobacco plants (Fig. 7G–K). In support of this, four AAP (general amino acid permeases) genes (*CL4328.Contig3*, *CL18378.Contig2*, *CL18707.Contig4* and *CL20735.Contig1*) and four GAT (GABA transporters) genes (*CL346.Contig8*, *Unigene346*, *Unigene347* and *Unigene87106*) were highly up-regulated during leaf senescence, especially in LL (Supplementary Fig. S10E–L). The transcripts for biosynthetic enzymes of these amino acids such as cysteine synthase (CysK) (*CL13724.Contig1*), however, was highly up-regulated in LL but stayed low in ML and UL (Supplementary Fig. S10M). These findings suggested that the high levels of the above-mentioned amino acids at upper leaf positions were at least partially the result of long distance transportation from lower senescing leaves. It seemed that active recycling of some of these amino acids happened at early stages of leaf senescence.

Besides primary metabolites, nicotine is an example of secondary metabolite being transported to organs distant from the sites of biosynthesis. In this study the changes of nicotine contents at different leaf positions supported the idea that higher nicotine accumulation in upper leaves being the result of transportation from lower parts of the plant, including the root system and senescing lower leaves (Fig. 7L). This is also supported by the up-regulation of the nicotine transporter genes (NUP and JAT1) in leaves at all positions, with greater changes in upper and middle leaves (9.5- and 2.9- fold changes for NUP and JAT1 in UL; 4.3- and 5- fold changes in ML, respectively;) compared to that in LL (3.2- and 2.1- fold changes for NUP and JAT1, respectively) (Fig. 6C). Furthermore, our results also suggested that a number of secondary metabolites seemed to be transported from senescing leaves at lower positions to sink leaves at upper positions. This included 2-furoic acid, nicotinic acid, caffeic acid, chlorogenic acid, 4-hydroxy-3-methoxybenzoic acid, hydroquinone, m-hydroxybenzoic acid, 4-hydroxybenzoic acid, guanosine, and uracil, some of which could play a role in nutrient remobilization (Supplementary Fig. S9). As an example, uracil was only found to be accumulated in upper and middle leaves. The uracil biosynthetic enzymes including asuridine nucleosidase (URH1) (*CL7700.Contig2*) and dihydropyrimidine dehydrogenase (DPYD) (*CL9191.Contig1*), however, were up-regulated at lower but not higher leaf positions (Supplementary Fig. S10N,O). We thus speculated that the specific distribution patterns of metabolites at different leaf positions were the results of long-distance transportation rather than local biosynthesis.

## Conclusion

Leaf senescence is a complex process. With the help of systems biological approaches, more and more data from high throughput studies are unraveling even more complexity of this process. Here we report the study of leaf senescence in which trancriptomic and metabolomic data are integrated, aiming to reveal a global picture of nutrient remobilization events. The sink-source relationship between leaves at different positions on the same plant makes tobacco a nice system for studying nutrient remobilization during leaf senescence. Spatiotemporal changes of 76 metabolites from leaves at three different stalk positions and eight developmental stages were compared with RNA-seq data. Metabolic pathways were analyzed via combining changes of related metabolites with the expression profiling of genes encoding for biosynthetic enzymes/transporters. We have identified a number of metabolic processes that showed changes in activities during leaf senescence. Particularly, we found a significant up-regulation of the TCA cycle and related metabolism of sugars, amino acids and fatty acids, supporting the importance of energy metabolism during leaf senescence. We also found strong evidence for the remobilization of nitrogen and nicotine during senescence. We have identified a number of compounds, including primary metabolites and secondary metabolites, which seemed to be transported from senescing leaves at lower positions to sink leaves at upper positions. Overall, our results provided new information in understanding the complexity of metabolic changes and nutrient remobilization during leaf senescence. Some of these findings could potentially provoke in depth investigations on the metabolic flux between senescing leaves and sink tissues.

## Methods

### Plant materials, growing conditions, and sampling

Plants of common tobacco (*Nicotiana tabacum* L.) variety Zhongyan100 were grown in the field (Zhucheng, Shandong, China) during the 2013 growing season. Upper, middle and lower positions Leaves of the same plant were collected for analysis at different developmental stages. Leaves at the 16^th^ leaf position from the bottom of the plants were defined upper leaves (UL). Leaves at the 9^th^ to 10^th^ positions were defined middle leaves (ML) and leaves at the 4^th^ position were named lower leaves (LL) in this study. The plants were grown and managed following regular practices and topping was done 60 days after transplanting when most of the plants were at early blossoming stages. Collection of leaf samples was done at 8 different time points during the growing season, started from 15 days after topping (15DAT). The sampling was carried out in the morning (9:00~10:00 am) once every 10 days. At each sampling time one leaf from each position (UL, ML, or LL) was collected from 12 plants. The plants were randomly selected and each selected plant was harvested only for one single time point to avoid effects from stress responses. For the UL and ML positions, eight samplings were done with the last sampling time being 85DAT. For the LL position, leaves were only collected four times because after 45DAT most leaves at this position were desiccated as a result of senescence (Fig. 1A). All harvested leaves were wrapped in aluminum foil, immediately placed in liquid nitrogen and stored at −80 °C until used. Immediately after harvesting each leaf was split into two equal parts along the mid vein: one of the halves was used for metabolomic analysis and the other half for transcriptomic analysis.

### Chlorophyll extraction and quantification

Chlorophyll was extracted and quantified as described^85^. Briefly, ten mg of freeze-dried leaf was extracted for 24 h with 1 ml of 95% ethanol in darkness with agitation. The supernatant was quantified via spectrophotometric determination at 649 and 665 nm. Chlorophyll contents were calculated and mean values from four biological replicates were obtained.

### Total protein measurement

Ten mg of freeze-dried ground material was homogenized in 1 ml extraction buffer containing 250 mM potassium phosphate (pH8.0), 0.5 mM EDTA, and 10 mM β-mercaptoethanol. The amount of soluble proteins in each sample was determined using Bradford reagent (Solarbio life sciences) according to the manufacturer’s instructions. Mean values from four biological replicates were obtained.

### Metabolite extraction and analysis

For each leaf position at each time point, samples from three different plants were mixed for metabolic analysis. For GC-MS analysis of most (70) of the metabolites, about 6 mg freeze-dried leaf power was extracted as reported^86,87^ with minor modifications. 1 μL of the extracted sample was analyzed by an Agilent 7890A-5975C GC/MS system (Agilent Technologies, USA). Metabolites were separated on a DB-5 MS column (30 m × 0.25 mm × 0.25 μm) with a split ratio of 10:1. The flow rate of carrier gas (99.9996% helium, Beijing, China) was maintained at 1.1 mL/min. The initial oven temperature was held at 60 °C for 1 min, then ramped by 10 °C/min to 325 °C, and kept for 10 min. The inlet temperature was set at 250 °C. The ionization was processed in EI positive mode. The temperatures of ion source and quadrupole were set at 230 °C and 150 °C, respectively. The mass spectra were acquired at a scanning range of 50–600 m/z.

For GC/MS analysis of anatabine and myosmine, an Agilent 7890 A gas chromatograph coupled with an Agilent 5975 C mass selective detector (Agilent Technologies, USA) was used according to the method of Xu *et al*.^88^. About 0.3 g freeze-dried leaf power was extracted. The initial oven temperature was set at 80 °C for 4 min, then increased at the rate of 10 °C/min to 200 °C (maintained for 20 min), and finally increased at the rate of 20 °C /min to 280 °C (maintained for 5 min). The injector, interface and ion source temperatures were set to 230 °C, 280 °C and 200 °C, respectively. The ionization mode was EI at 70 eV. The MS detector was operated in scan mode (mass range 35–300 amu).

The concentrations of neochlorogenic acid, rutin, scopoletin and solanesol were determined by high performance liquid chromatography using a ACQUITY UPLC H-Class system (Waters Corp., Milford, MA, USA) according to the method of Duan *et al*.^89^. The quantitative analysis of the components was achieved with reference to the authentic standards neochlorogenic acid, rutin, scopoletin and solanesol, which were purchased from Sigma-Aldrich (St. Louis, MO, USA).

Mean values from four biological replicates were obtained. For GC/MS analysis, In addition to identifying compounds based on mass spectral search against the NIST (the National Institute of Standards and Technology) 2008 spectral library (similarity factor set at 800), the identities of 35 compounds were further verified with reference standards (detail information shown in Supplementary Table S5).

### RNA extraction, library construction and RNA-seq

Leaf samples from six different plants were mixed to represent each leaf position at each time point and two biological replicates were used for RNA-seq analysis. Total RNA was extracted following the previously described method^85^. The quality and concentration of extracted RNA were determined using a Nanodrop 2000 spectrophotometer (Thermo Scientific, Wilmington, USA) and an Agilent 2100 Bioanalyzer. Message RNA purification, library preparation and RNA-seq were performed by Beijing Genomics Institute (BGI). For each sample, DNase treatment of total RNA was firstly performed using deoxyribonuclease I (DNase I). Then, poly (A)-containing mRNA molecules were purified from total RNA using poly (T) oligo-attached magnetic beads. After that mRNA was fragmented into small pieces using the RNA fragmentation kit at elevated temperatures in Thermomixer, followed by first- and second-strand cDNA synthesis using random hexamer primers. Finally, after the operation of ends reparation, A-Tailing, adaptor ligation and products purification, short cDNA libraries were prepared for sequencing according to manufacturer’s protocol. In total, forty libraries were obtained and sequenced using an Illumina HiSeq™ 2000 sequencer. The whole set of original sequences have been deposited in the NCBI Sequence Read Archive under accession numbers SRP102153.

### *De novo* assembly, function annotation and RNA-seq data analysis

The transcriptome of tobacco leaf senescence was analyzed via *de novo* assembly of the RNA-Seq data. Firstly, the raw reads were filtered by removing adaptor sequences, reads with more than 5% unknown nucleotides, and low quality reads containing more than 20% of bases with quality value <= 10. Then, these clean reads were assembled to the longest assembled sequences (called contigs) using Trinity by BGI^90^. The reads were mapped again back to contigs and get sequences that have least Ns and cannot be extended on either end (called unigenes). Totally, forty unigene databases were obtained for all samples. Finally, all the unigenes were assembled from 40 samples to form a single set of non-redundant unigenes using TGICL^91^.

The unigenes were analyzed by searching various databases such as nt, nr, KEGG, Swiss-Prot and COG using BLAST (E-value < 1e-5) to predict their functions. Unigenes with an nr annotation were further analyzed with the Blast2GO (v2.5.0) software to obtain GO annotations, and were further classified according to GO functions using WEGO^92,93^.

### Identification of differentially expressed genes (DEGs)

SOAPaligner (Version 2.21) was used to map reads back to the unigenes at the parameters of “-m 0 -x 500 -s 40 -l 35 -v 5 -r 1” and the number of mapped reads for each unigene was recorded. Then expression profiles of the unigenes were analyzed with the FPKM method. In this study, DEGs between each of the samples were defined to have a threshold of false discovery rate (FDR) <= 0.001 and an absolute value of Log2 ratio >= 1. Furthermore, the GO classifications were compared between up-regulation and down-regulation unigenes using WEGO, using the corrected-p value <= 0.05 as a threshold.

### Metabolic change and pathway enrichment analysis

To identify metabolic changes between different developmental stages, the metabolite contents of leaves from each developmental stage at the same position were normalized with the first stage (15DAT) as a reference point. Changes were shown in false color by Log2 fold changes of metabolite levels for a better comparison and visualization of the measured differences.

Metabolic pathway enrichment analysis was performed for the DEGs compared to the whole transcriptome background. First, the Blastall program was used to annotate the pathways of DEGs against the KEGG database. Subsequently, enriched pathways of DEGs were identified using hypergeometric test with Q-value <= 0.05. Moreover, Interactive pathways (ipath) analysis was carried out via the Interactive Pathways Explorer (version 2.0) (http://pathways.embl.de/). Through KO (KEGG Orthology) id, three different colors were applied to indicate different changing patterns of pathways and genes, with red color for up-regulationg, green color for down-regulation, yellow for irregular regulation.

### Real-time PCR validation

First-strand cDNA synthesis was performed with 4 μg of total RNA using the Revert Aid First Strand cDNA synthesis kit (Thermo Fisher Scientific) according to the manufacturer’s instructions. The transcriptional profiles of selected genes were analyzed via real-time RT-PCR using the FS Universal SYBR Green master mix (Roche) and a 7500 real-time PCR system (Applied biosystems). The PCR involved 95 °C hold for 10 min followed by 40 cycles at 95 °C for 15 s and 60 °C for 35 s. The relative expression ratio values for different development time points relative to the first sampling time point (15DAT) were calculated using 2^−ΔΔCT^ method. The Actin gene of *N. tabacum* was used as a reference gene for normalization of the expression. The primers G0571 (5′-ACCTCTATGGCAACATTGTGCTCAG-3′) and G0572 (5′-CTGGGAGCCAAAGCGGTGATT-3′) were used for Actin7 gene qRT-PCR assay. The primers G0585 (5′-CAGTGGCTAATCAACCTGTTTCGG-3′) and G0586 (5′-ACACCACTTGAATAGAACTGGAAATCG-3′) were used for CP1 gene qRT-PCR assay. The primers G0587 (5′-CGAAACTCTCTCATACCTTCCCGA-3′) and G0588 (5′-CATGGTCCAGTATCTGCCGTCATA-3′) were used for RBCS gene qRT-PCR assay.

## Electronic supplementary material

Supplementary Figure S1-S10

Supplementary Table S1

Supplementary Table S2

Supplementary Table S3

Supplementary Table S4

Supplementary Table S5

Supplementary Table S6
