# Supplementary material for: Intergrative metabolomic and transcriptomic analyses unveil nutrient remobilization events in leaf senescence of tobacco
Source: Sci Rep. 2017 Sep 21;7:12126. doi: 10.1038/s41598-017-11615-0 (PMC5608745; doi:10.1038/s41598-017-11615-0)
Supplement: Supplementary file 1 — Supplementary Figure S1-S10 [file 41598_2017_11615_MOESM1_ESM.pdf]

Supplementary Information

Intergrative Metabolomic and transcriptomic analyses unveil nutrient remobilization events in leaf senescence of tobacco

Wei Li[1], Hailiang Zhang[1], Xiaoxu Li[1], Fengxia Zhang[2], Cheng Liu[1], Yongmei Du[1], Xiaoming Gao[1], Zenglin Zhang[1], Xiaobing Zhang[1], Zhihui Hou[1], Hui Zhou[1], Xiaofei Sheng[1], Guodong Wang[2], Yongfeng Guo[1,*]

[1] Tobacco Research Institute, Chinese Academy of Agricultural Sciences, Qingdao, Shandong 266101, China

[2] Institute of Genetics and Developmental Biology, Chinese Academy of Sciences, Datun Road, Chaoyang District, Beijing 100101, China

[*]Corresponding author: Yongfeng Guo, Email: guoyongfeng@caas.cn, Tel: +86-532-66715256

Number of supplementary figures: 9

Number of supplementary tables: 6

Figure S1

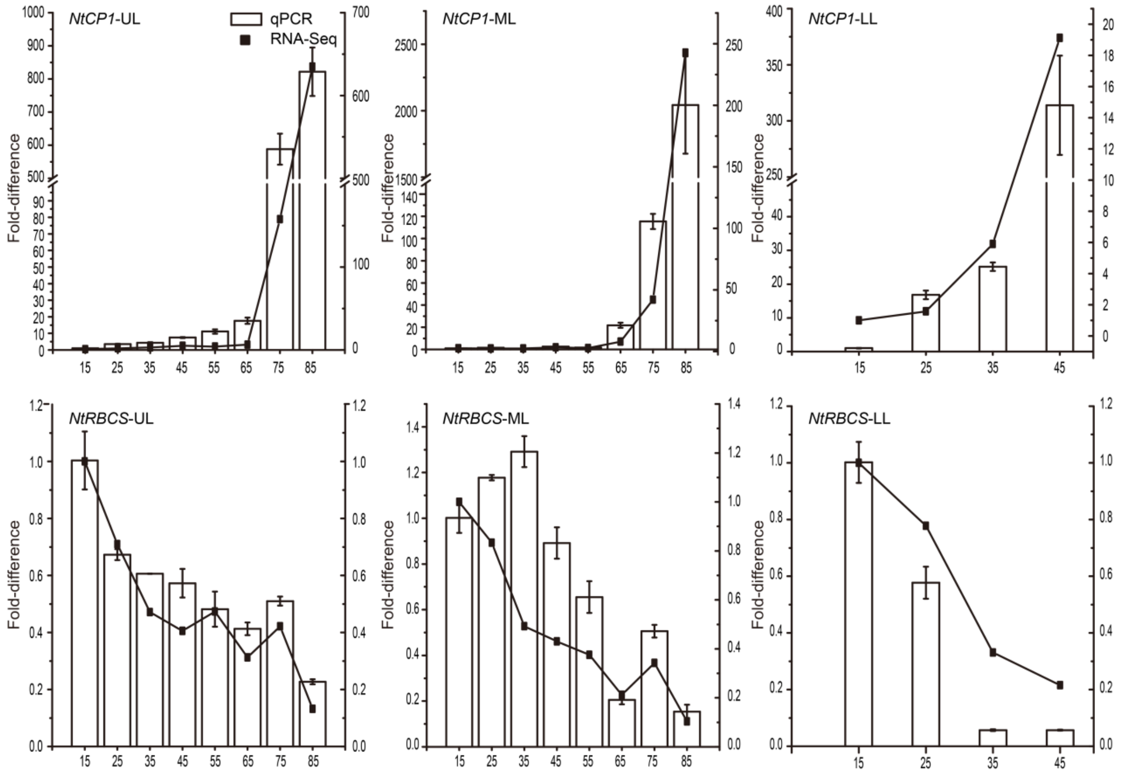

Figure S1. Validation of RNA-Seq expression profiles using realtime quantative PCR (qPCR). Bars and lines depcit expression changes of senescence marker genes (*NtCP1* and *NtRBCS*) in qPCR and RNA-Seq data from 15 to 85DAT, respectively. UL, upper leaf; ML, middle leaf; LL, lower leaf.

Figure S2

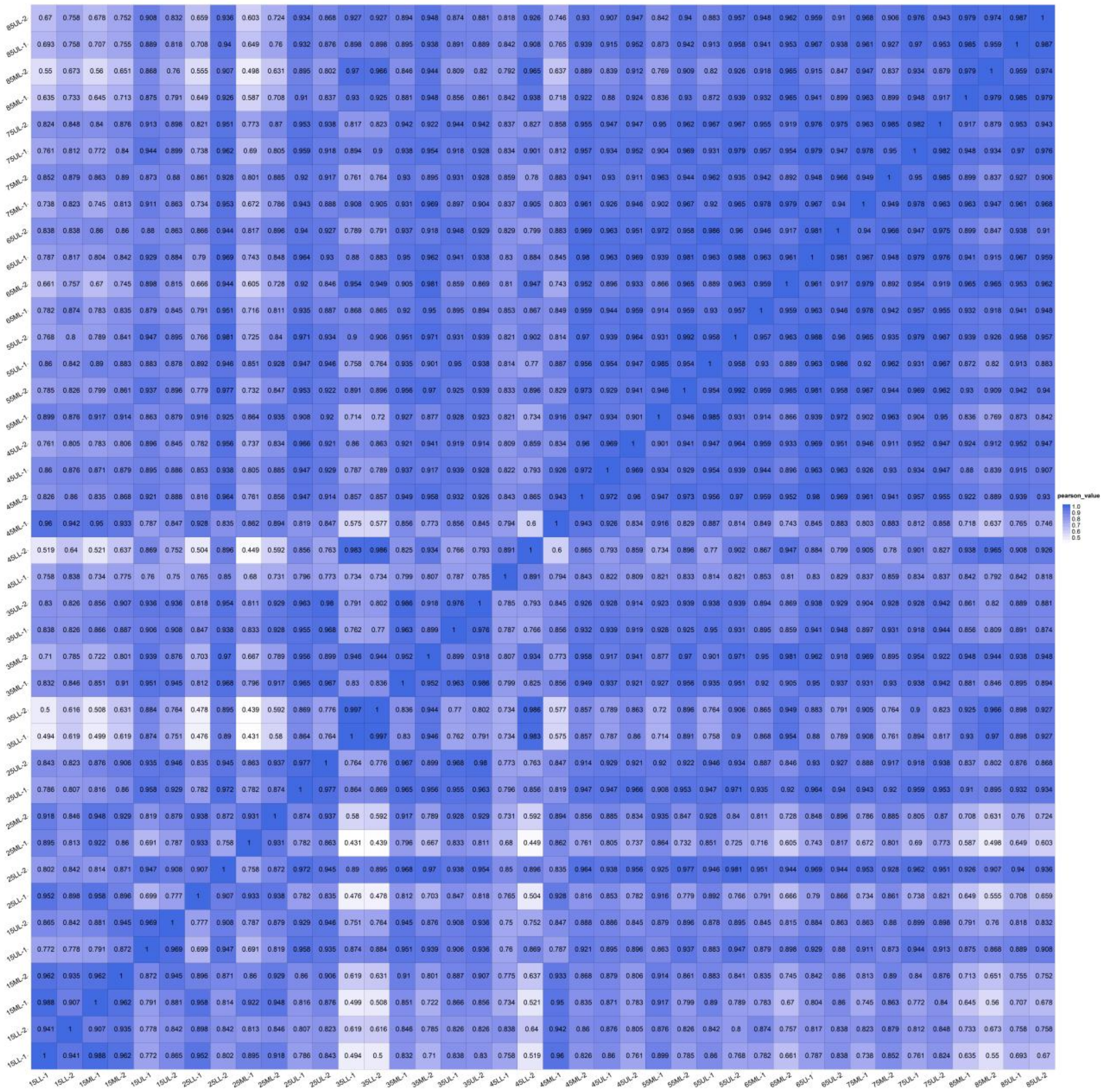

Figure S2 Pairwise correlation between samples measured by RNA-Seq.

Figure S3

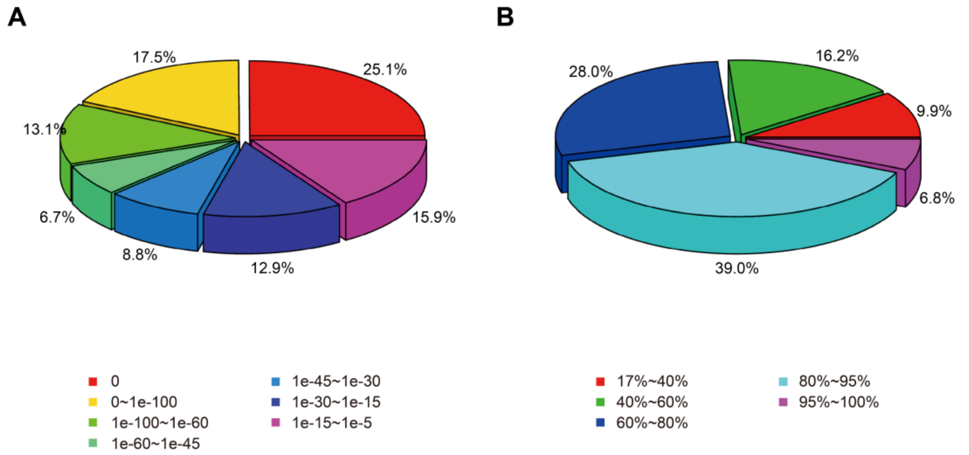

Figure S3 NR classification of tobacco unigenes. (A) The E-value distribution of the result of NR annotation. (B) The similarity distribution of the result of NR annotation.

Figure S4

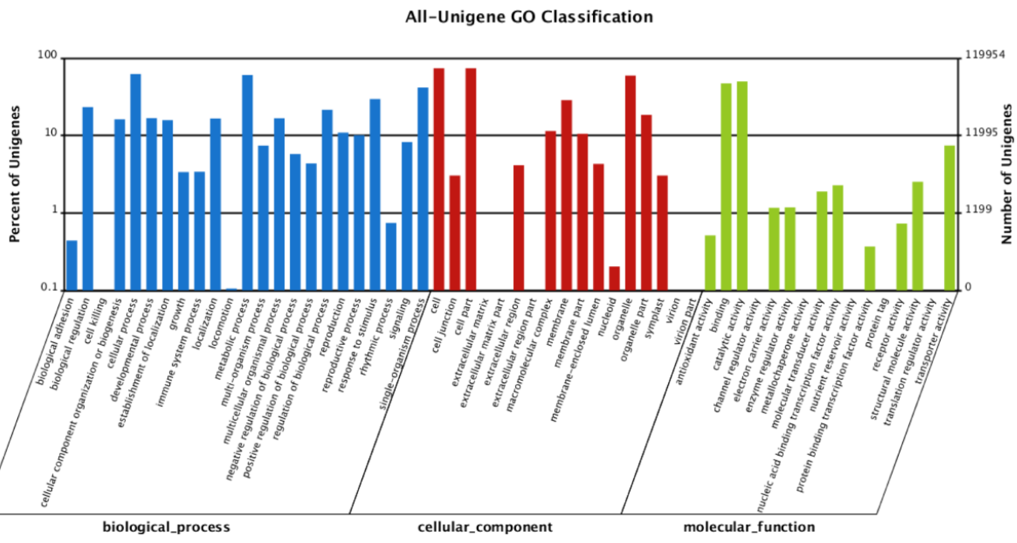

Figure S4 GO classification of unigenes in tobacco.

Figure S5

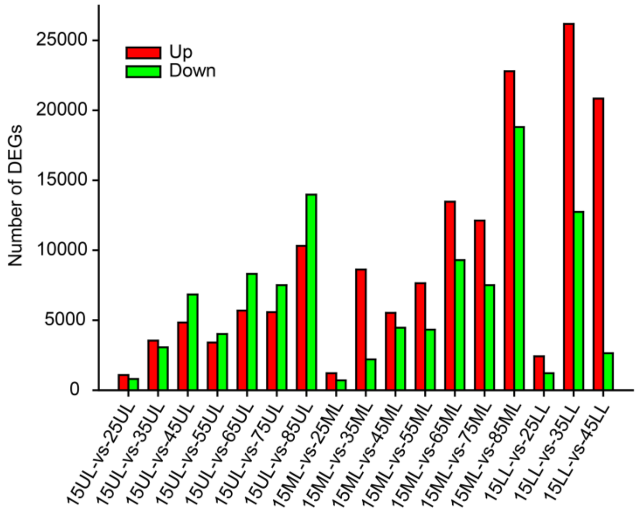

Figure S5 Numbers of differentially expressed genes (DEGs) identified by pair comparison between different development stages.

Figure S6

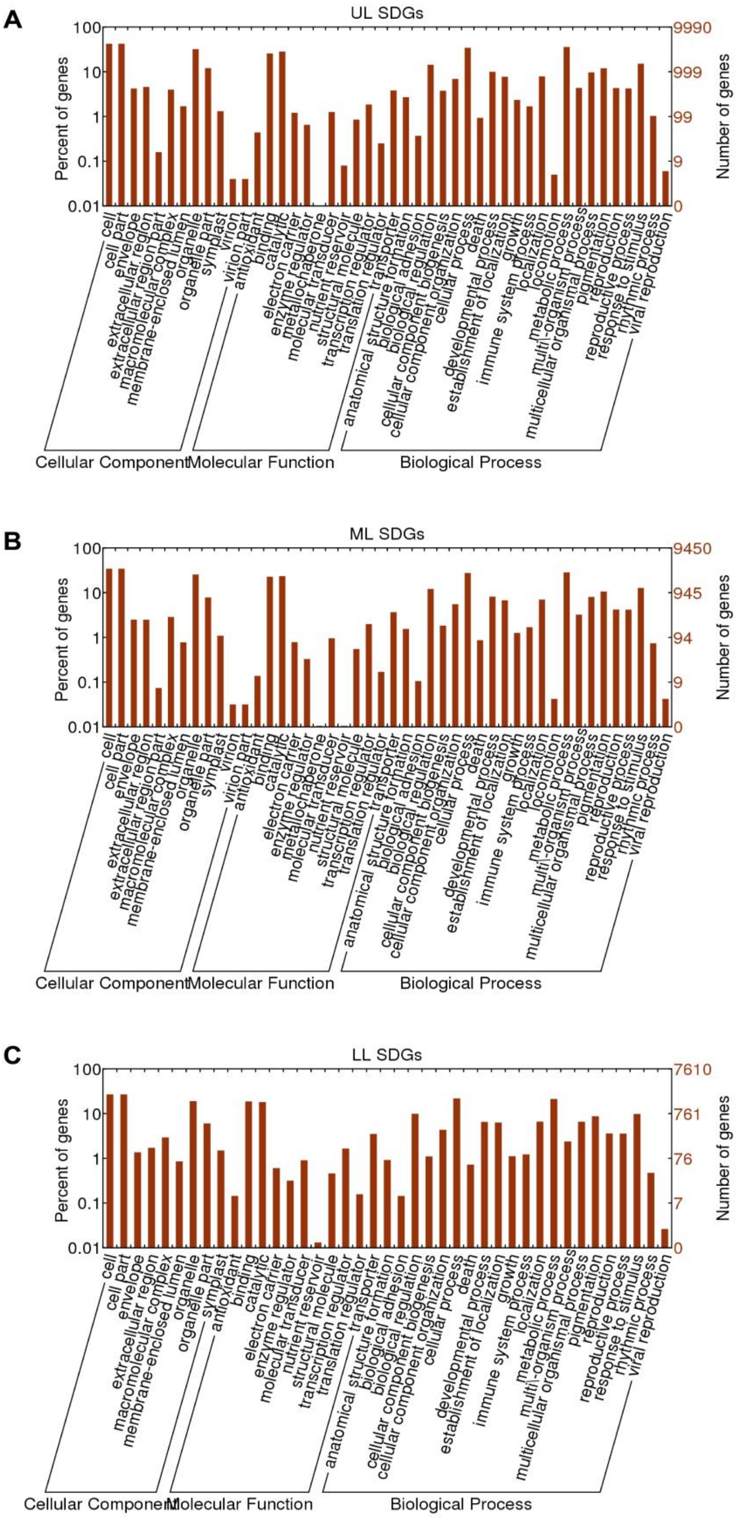

Figure S6 GO classification of SDGs in UL, ML and LL during tobacco leaf senescence. (A) Upper leaf (UL). (B) Middle leaf (ML). (C) Lower leaf (LL).

Figure S7

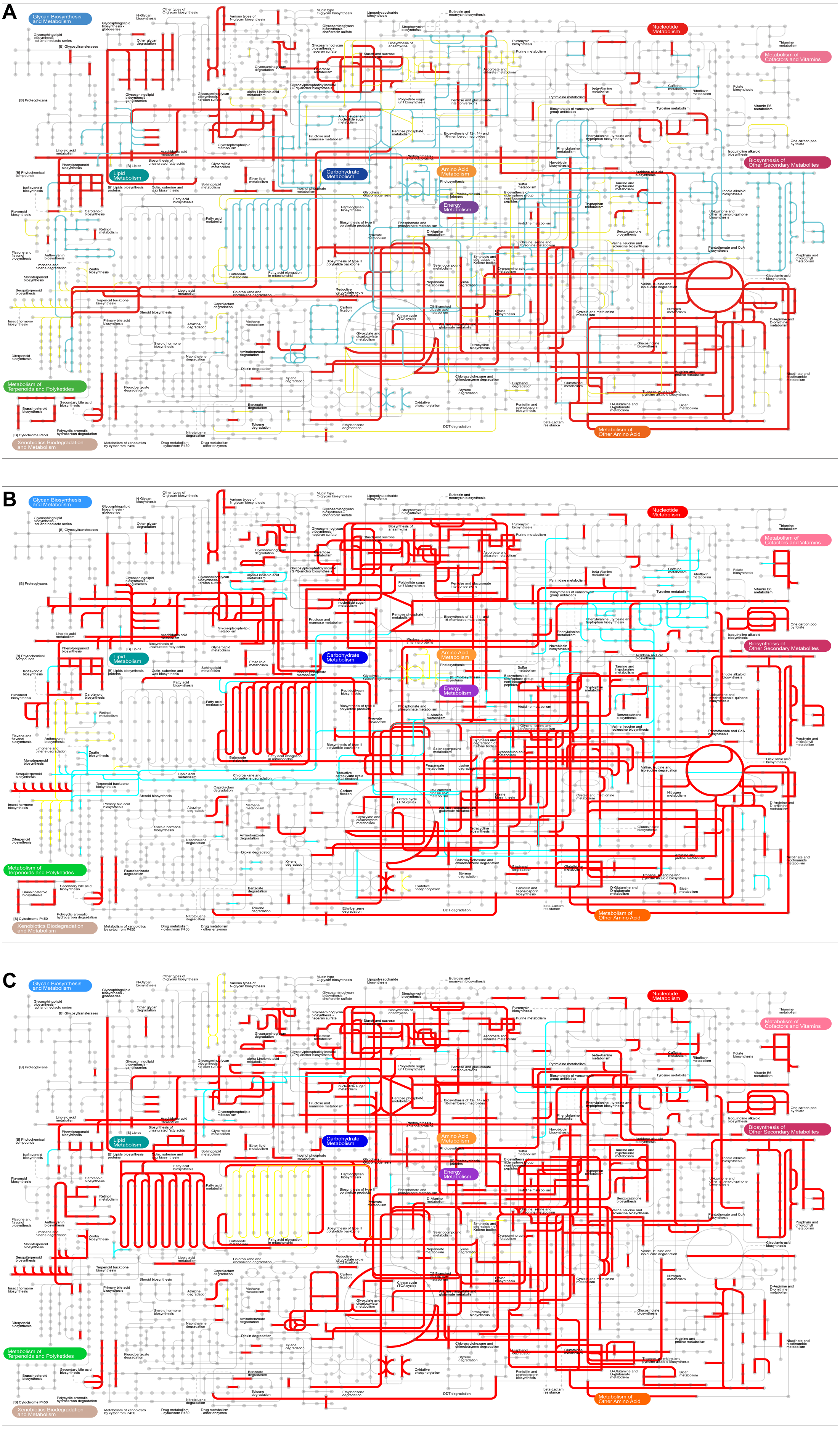

Figure S7 Interactive pathway analysis of DEGs in UL, ML and LL during tobacco leaf senescence. (A) Upper leaf (UL). (B) Middle leaf (ML). (C) Lower leaf (LL). The red, the green, the yellow lines indicate metabolic pathways with up-regulated, down-regulated and irregularly regulated, respectively.

Figure S8

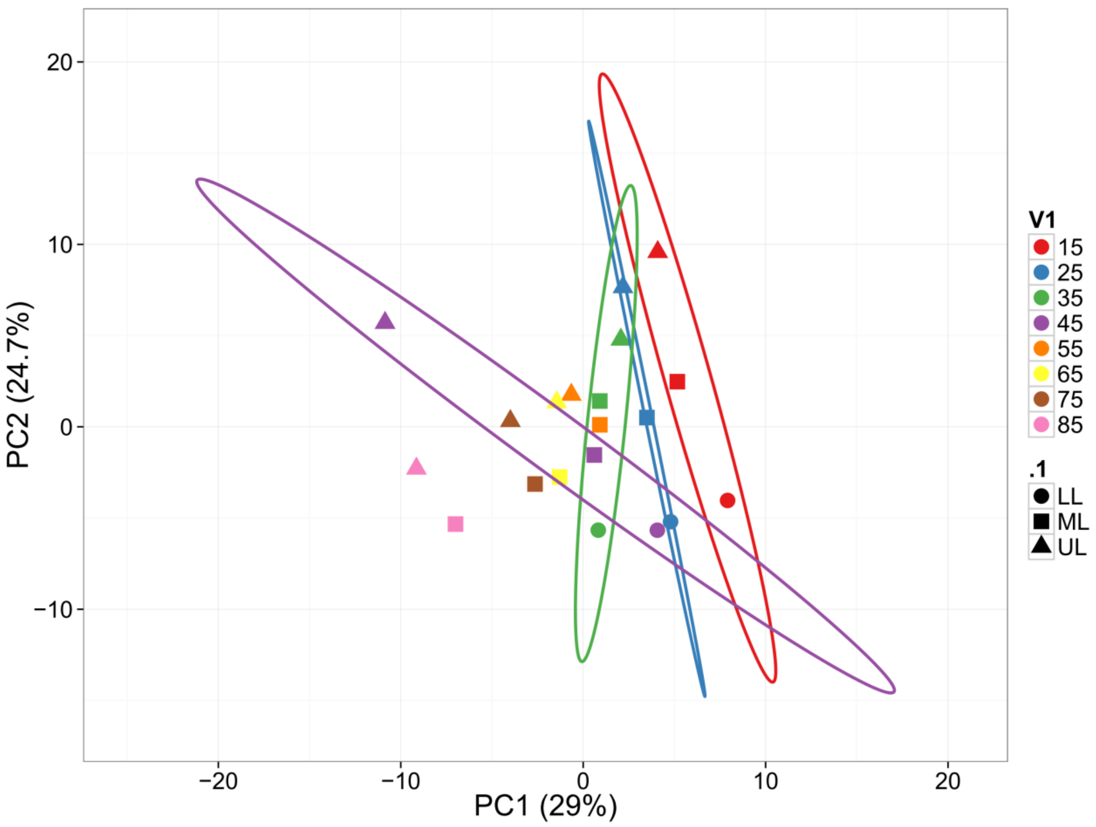

Figure S8 Principal component analysis (PCA) of metabolite profiles for all the position leaves during tobacco leaf senescence. Each point represents an mean value of four biological replicates. Red, blue, green, purple, orange, yellow, brown and pink colors represent samples at 15, 25, 35, 45, 55, 65, 75 and 85DAT, respectively. Triangle, box and dot denote metabolomes of UL, ML and LL, respectively. Plotting of the first and second component is shown. The circles indicate the 95% confident regions.

Figure S9

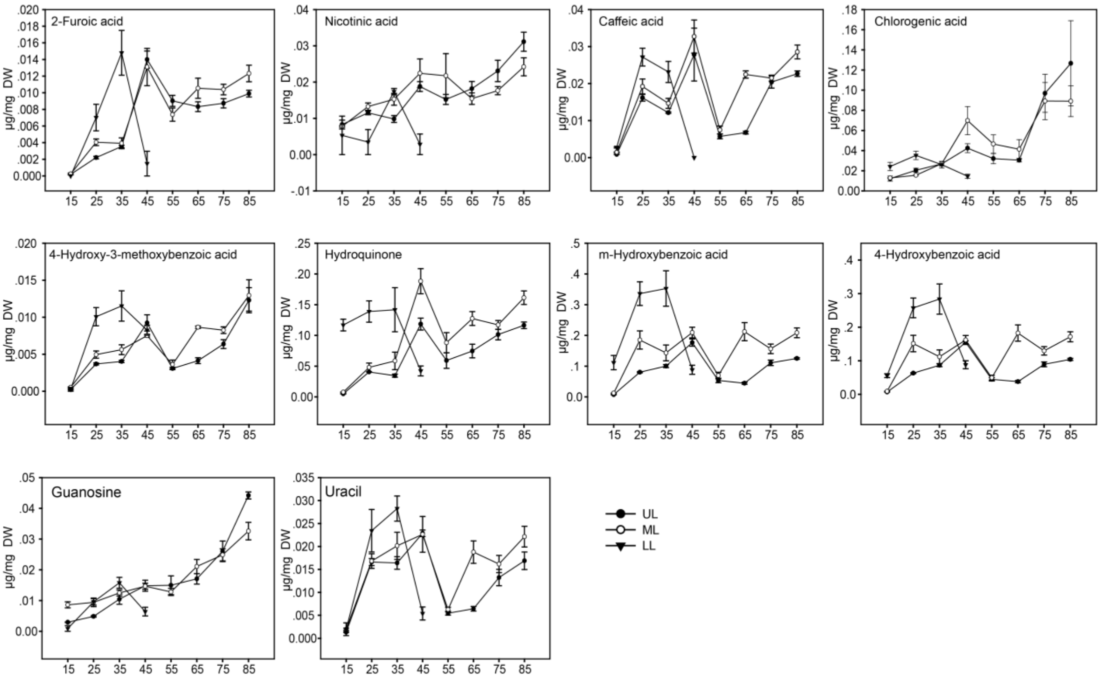

Figure S9 Relative contents of metabolites which seem to be transported from senescing leaves to sink leaves.

Figure S10

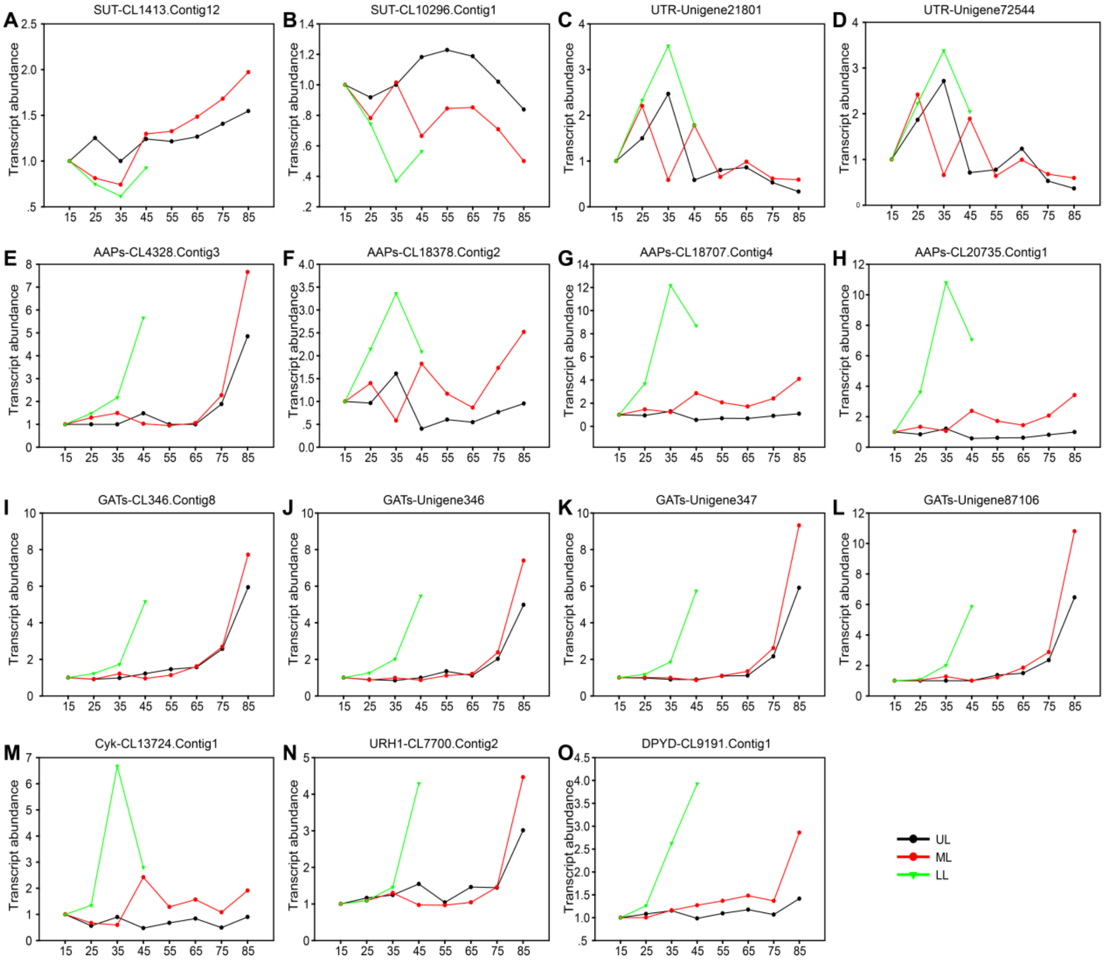

Figure S10 Expression patterns of transporter and biosynthesis genes of the selected metabolites which are candidate metabolites for long distance transportation during senescence.
